# Trithiocarbonate-Functionalized PNiPAAm-Based Nanocomposites for Antimicrobial Properties

**DOI:** 10.3390/polym10060665

**Published:** 2018-06-14

**Authors:** Milène Tan, Lenke Horvàth, Priscilla S. Brunetto, Katharina M. Fromm

**Affiliations:** Department of Chemistry, University of Fribourg, Chemin du Musée, 9, 1700 Fribourg, Switzerland; milene.tan@unifr.ch (M.T.); lenke.horvath@unifr.ch (L.H.); priscilla.brunetto@unifr.ch (P.S.B.)

**Keywords:** silver, antimicrobial, poly(*N*-isopropylacrylamide), nanocomposites

## Abstract

In this study, four trithiocarbonate-functionalized PNiPAAms with different molecular weights were synthesized and used as a matrix to form composites with silver nanoparticles. Nanocomposites with several polymer-to-silver ratios P:Ag^+^ were prepared in order to evaluate the influence of silver loading. UV studies showed a thermoresponsive behavior of the nanocomposites with a thermo-reversibility according to cooling-heating cycles. Release kinetics demonstrated that the release of silver ions is mainly influenced by the size of the silver nanoparticles (AgNPs), which themselves depend on the polymer length. Antimicrobial tests against *E. coli* and *S. aureus* showed that some of the nanocomposites are antimicrobial and even full killing could be induced.

## 1. Introduction

The increased use of medical devices coupled with the emergence of antibiotic resistant bacteria has caused a serious healthcare concern for biomaterials-related infections. Despite sterilization procedures, these devices can still become contaminated by bacteria, and once implanted into a patient, they may lead to infection, sometimes resulting in death [1,2,3]. Many strategies have been employed to limit these cases. One of the most promising is the use of silver-based compounds such as coordination compounds [4,5], coordination polymers [6,7], silver-filled nanocontainers [8,9], and silver polymer composites [10,11,12]. The silver ion has been long known to possess antibacterial properties, even at low concentrations (0.1–10 ppm) [13]. Interestingly, silver nanoparticles (AgNPs) are amongst the most efficient combatants against bacteria, maintaining a longer-lasting activity (up to 100 days) [14,15]. Due to the multidirectional targets of silver, the bacteria are usually less susceptible to developing a resistance against silver as compared to antibiotics [16,17]. However, the utilization of AgNP-containing materials still faces many challenges, including issues of stabilization, aggregation, and release control of the silver ions [18,19,20,21]. One of the most promising strategies to overcome these impediments is the use of polymers to form functional polymer/silver nanocomposites [22,23]. These polymers show many advantages. They can be used as surrounding media during particle synthesis, leading to better stabilization of the AgNPs [24,25]. They allow dispersion of the nanoparticles, preventing aggregation, which is shown to reduce antibacterial activity [26,27]. Finally, they can be easily functionalized to provide binding sites for silver, leading to immobilization of the nanoparticles. Fixing the particles in the matrix is key to restricting the cell uptake, thus diminishing the cytotoxicity. These polymers can also be engineered to play a role in the kinetic of silver ion release.

Stimuli responsive polymers in particular are very promising candidates for controlling silver ion release. By definition, upon stimulus, these polymers undergo a physical or chemical change. An example that has been thoroughly studied is Poly(*N*-isopropylacrylamide) (PNiPAAm), a well-known thermoresponsive polymer [28,29,30]. This material is characterized by a low critical solubility temperature (LCST), close to 32 °C in water. Practically, below 32 °C, the polymer has an expanded coil state and is hydrophilic. While above the LCST, the polymer changes to a collapsed globular state and becomes hydrophobic [31,32,33,34,35]. This behavior is very interesting for delivery applications since the collapsed phase of the polymer can distribute potential medicines [36,37,38]

Elashnikov et al. investigated the properties of Poly(methylmethacrylate) PMMA-/PNiPAAm/Ag films. Using the diffusion method, they obtained an antimicrobial response against *S. aureus* and *P. aeruginosa.* However, they did not study the thermoresponsive behavior of their films [32]. Spasojevic et al. studied the formation of AgNPs in a PNiPAAm-based hydrogel/xerogel using gamma irradiation [39], focusing on the AgNP morphology, size, and bonding environment, as well as the structure of the final composites. He et al. studied the behavior of thermoresponsive hydrogel loaded with AgNPs [40]. Their AgNP-hydrogels, based on PNiPAAm and sodium acrylate (AANa), possess antimicrobial properties and low cytoxicity, but were not investigated for the influence of their molecular weight during the formation of the AgNPs. In addition, they did not study neither the stability of their composites, nor the effect of AgNPs on their physicochemical and thermal properties such as the LCST [40]. 

Here, we chose to prepare trithiocarbonate-functionalized PNiPAAms (TTC-PNiPAAms) for specific silver ion binding and to investigate the influence of molar mass, temperature, and silver content on the ability of these TTC-PNiPAAms to release silver ions in a controlled manner. The thermal properties were measured to evaluate the influence of the AgNPs on the polymers. Antimicrobial properties and cell viability of the nanocomposites were also studied. These composites could be further used as potential coatings for medical devices to prevent the infection and biofilm formation. 

## 2. Materials and Methods 

### 2.1. Materials

All polymerization reactions were carried out under an inert argon atmosphere using the Schlenk line technique. Prior to polymerization, *N*-isopropylacrylamide (NiPAAm, 99%, Acros, Geel, Belgium) and 2,2′-Azobisisobutyronitrile (AIBN, 98%, Sigma-Aldrich, Steinheim, Germany were recrystallized twice in n-hexane and methanol, respectively. 2-(Dodecylthiocarbonothioylthio)propionic acid (DMP, 97%, Aldrich, Buchs, Switzerland), silver nitrate, and ammonia (28–30%, Acros, Geel, Belgium) were used as received.

### 2.2. Synthesis of Trithiocarbonate-Functionalized Poly(N-Isopropylacrylamide) (TTC-PNiPAAm) by RAFT Polymerization

TTC-PNiPAAm with a targeted degree of polymerization (DP) of 25, 50, 100, and 200, respectively, were synthesized. In a typical procedure, TTC-PNiPAAm with a targeted degree of polymerization (DP) of 25 (polymer **A**) was prepared as follows: In a Schlenk flask, NiPAAm (6 g, 53.6 mmol), 2-(Dodecylthiocarbonothioylthio)propionic acid (DMP) (0.772 g, 2.12 mmol), and 2,2′ azobisisobutyronitrile (AIBN) (0.069 g, 0.42 mmol) were dissolved in tetrahydrofuran (THF) (18.57 mL). The flask was purged by three cycles of freeze-pumping-thawing in order to remove all oxygen. The polymerization was then conducted under argon atmosphere at 70 °C. After 2 h, the polymer was slowly precipitated in cold diethyl ether (seven volumes). This step was repeated twice to yield a yellow powder. SEC (UV): 1.1, ^1^H-NMR (CDCl_3_, 400 MHz): δ 0.87 (t, 3H, *CH_3_*–) 1.13 (m, 6H, (*CH_3_*)_2_–CH–NH–), 1.25 (s, 18H, CH_3_–(*CH_2_*)_9_–CH_2_–), 1.38 (s, 2H, CH_3_–(CH_2_)_9_–*CH_2_*–CH_2_–S–), 1.63 (s, 6H and 2H, (*CH_3_*)_2_–C(COOH)–CH_2_) and S–CH–*CH*_2_–C*–*), 2.12 (s, 1H, S–*CH*–CH_2_–), 3.32 (m, 2H, CH_2_–*CH_2_*–S–C(S)–S–), 3.99 (s, 1H, NH–*CH*–(CH_3_)_2_), and 6.47 (s, 1H, C(O)–*NH*–CH–). The ppm shifts do not change for the other polymers as the molecular weight increases. The NMR spectra for all the polymers are in the supporting information Figures S1 to S4.

### 2.3. Synthesis of the Nanocomposites

For each polymer, nanocomposites at four different ratios of polymer to Ag^+^ were prepared (Table 1). AgNPs were synthesized following the procedure developed by Niitsoo et al. [41]. Typically, silver nitrate (AgNO_3_) (66.3 mg, 0.39 mol) was dissolved in water (9.45 mL) and sodium hydroxide (NaOH) 1M (0.39 mL) was added to the solution to form silver dioxide (Ag_2_O). Then, ammonia (NH_4_OH) solution (0.16 mL of 28–30%) was added dropwise to form the Tollens reagent [Ag(NH_3_)_2_]^+^. A total of 1 mL of this solution was added to a solution containing the TTC-PNiPAAm (quantity according to P:Ag^+^ in molar ratio) and glucose (0.234 mL of 300 mg/10 mL) as the reducing agent. The polymer allows the stabilization of the nanoparticles and prevents the aggregation. The mixture was stirred for 5–15 min at 750 rpm at 25 °C. The solution was then frozen with liquid nitrogen and dried under vacuum for 24 h. 

### 2.4. Characterization

Nuclear Magnetic Resonance (NMR) spectra were recorded on a Bruker Avance III spectrometer (Bruker, Fällanden, Switzerland) at 400 MHz in CDCl_3_. Size exclusion chromatography (SEC) measurements were carried out on an Agilent Technologies 1200 system equipped with a Wyatt Optilab rEX differential refractive index (dRI) detector and a Wyatt miniDAWN TREOS multiangle laser light scattering (MALLS) detector (Agilent, Basel, Switzerland). The column system was composed of an Agilent 5 μm MIXED-C guard column and either a PLgel 5 μm MIXED-C (200–2,000,000 g/mol) column or a 5 μm MIXED-D (200–400,000 g/mol) column from Agilent. THF was used as the solvent/eluent and the measurements were carried out at a flow rate of 1 mL/min. 

The morphology and the size of the AgNPs were determined by transmission electron microscopy (TEM) using a FEI/Philips CM 100 Biotwin transmission electron microscope (the operating voltage = 80 keV, in bright-field mode, FEI, Zürich, Switzerland). The size and the distribution of the nanoparticles were obtained by ImageJ. 

UV-vis spectra were recorded with a UV-vis Spectrometer (Lambda40, Perkin Elmer, Bahnstrasse, Schwerzenbach) at wavelengths ranging from 250 to 600 nm.

The thermogravimetric analysis (TGA) measurements were performed on a TGA/SDTA 851e (Mettler-Toledo, Bussigny, Switzerland). The measurements were done under air conditions with a rate of 5 °C/min from 25 to 500 °C, starting with an isotherm of 30 min at 50 °C. 

Differential scanning measurements (DSC) were carried out under N_2_ on Mettler-Toledo Stare DSC or DSC 2 systems (Mettler-Toledo, Bussigny, Switzerland) at heating and cooling rates of 10 °C/min. 

Powder X-ray diffractograms (PXRD) were collected on a Stoe StadiP (STOE, Darmstadt, Germany) using Cu K_α1_ radiation (1.5406 Å). 

### 2.5. Silver Loading 

Silver loading was determined by inductively coupled plasma optical emission spectroscopy (ICP-OES) using the PerkinElmer Optical Emission Spectrometer (Optima 7000 DV, PerkinElmer, Basel, Switzerland). A total of 1 mg of the nanocomposite sample was dissolved in concentrated nitric acid (65%) (1.54. mL) in order to destroy the polymer and to dissolve the nanoparticles. The sample was sonicated (Ultrasonic, TPC-25, *W*_max_ 150) for 30 min at room temperature and left overnight in 65% nitric acid. Finally, pure water was added to reach 10% of nitric acid. 

### 2.6. Lower Critical Solubility Temperature (LCST) Determination

The lower critical solubility temperature was determined by measuring the absorbance of the sample solutions (1 mg/mL in H_2_O) at 650 nm. The temperature was increased from 32 to 52 °C. The graphics were plotted as transmittance versus temperature. A loss of 50% of the transmittance corresponds to the cloud point. 

### 2.7. Stability

The stability of the nanocomposites (**3**) and (**4**) was determined in phosphate buffer (PBS, 0.01 M, pH = 7.4) at 37 °C. The solutions were analyzed by UV-vis spectroscopy over two weeks to observe the decrease of the AgNPs’ absorption band. 

### 2.8. Release Kinetics

The release was performed based on the principle of dialysis tubing [42]. The samples (in triplicate) were prepared at a concentration of 1 mg/mL (Polymers **A** and **B**) and at 2 mg/mL (Polymers **C** and **D**) in pure water. A total of 2 mL of the solution was placed in a dialysis bag (0.79 mL/cm, Cellulose ester MWCO 1 kDa, Spectra/Por, SpectrumLabs, Darmstadt, Germany) and 50 mL of water was added into the dialysis container. At different time intervals, the dialysate was removed and replaced by 50 mL of pure water. The silver concentration was determined by ICP-OES, and each time point was measured in triplicate. The release was performed at room temperature (RT) and 37 °C to see the effect of the temperature on the polymer.

### 2.9. Antimicrobial Tests

Bacterial strains and growth conditions:

Escherichia coli (*E. coli*) bacteria (ATCC^®^ 25922) were freshly grown in a Mueller Hinton broth (MHB) (Sigma-Aldrich, St. Louis, MO, USA) overnight at 37 °C with shaking (180 rpm). Bacterial numbers were estimated by determining the turbidity (McFarland) at OD_600_ [43]. 

*Staphylococcus aureus* (*S. aureus*) bacteria (113 wt) were freshly grown in Tryptic soy broth (TSB, Bacto^TM^ BD, Sparks, NV, USA) for 6 h at 37 °C with shaking (180 rpm). The broth was then diluted 1:100 in 10 mL MHB and left to grow overnight at 37 °C with shaking (150 rpm). 

Microbroth dilution assay: 

The antimicrobial tests were performed in a 96-well plate with a TECAN reader (TECAN, Männedorf, Switzerland). Practically, different concentrations (ranging from 0.25 mg/mL to 2 mg/mL) of the samples dissolved in Mueller Hinton broth, inoculated or not with bacteria *E. coli* or *S. aureus* (10^6^ CFU/mL), were put in a 96-well plate. The medium was used as the negative control. Optical density (OD) was taken at 600 nm each two hours for 24 h.

Graphics of the bacterial growth (OD_600_) with the nanocomposites were plotted as a function of time (hours). 

### 2.10. Biocompatibility

Cell culture:

Murine fibroblast cells L-929 (NCTC clone 929) were obtained from American Type Culture Collection (ATCC, Manassas, VA, USA, catalogue number: CCL-1). The cells were cultured in RPMI-1640, GlutaMAX^TM^ (Gibco, Zoug, Switzerland), supplemented with 10% heat-inactivated fetal bovine serum (PAN Biotech, Chemie Brunschwig, Auf dem Wolf, Basel) and antibiotics (10,000 U/mL penicillin, 10 mg/mL streptomycin; Gibco) and were maintained in a humidified atmosphere containing 5% CO_2_ at 37 °C. The medium was replaced every two to three days and cells were subcultured through trypsinization when reaching near-confluence. Initial cell concentrations were calculated using the Trypan blue exclusion method (0.4% Trypan blue solution, T8154; Sigma-Aldrich, St. Louis, MO, USA). The working cell concentrations were prepared by diluting cells with cell culture medium. 

Cell viability tests: 

L929-Fibroblasts cells were seeded in 96-well flat-bottomed tissue culture plates (TPP, Switzerland) at a concentration of 2 × 10^3^ cells/well (4 × 10^4^ cells per mL). After an overnight incubation (once adhesion was verified), 50 µL of fresh medium containing the corresponding amount of polymers was added to the cells. Cells were exposed to 1, 10, and 100 µg/mL polymers for 24 and 72 h. All parallel cultures serving as untreated controls were also supplemented with fresh medium and served as a reference for 100% cell viability. Each experiment was repeated at least three times with three replicates of the same material and concentration.

Cell viability was evaluated by the MTT (3-(4,5-dimethylthiazol-2-yl)-2,5-diphenyltetrazolium bromide) assay (thiazolyl blue tetrazolium bromide; M5655, Sigma-Aldrich, Buchs, Switzerland) reporting the combined effects of proliferation (number of viable cells) and cellular metabolic activity [44]. The assay is based on the accumulation of dark blue formazan crystals inside living cells after their exposure to MTT (10 μL of 5 mg/mL). After removal of the cell culture medium, dimethylsulfoxide (DMSO, Sigma-Aldrich, Steinheim, Germany, 100 μL/well, 30 min incubation at RT on orbital shaker) was added to wells, which permeabilizes the cell membrane and results in the liberation and solubilization of the formazan crystals. The formazan concentration was quantified using a multilabel plate reader (PerkinElmer VICTOR X3, PerkinElmer, Basel, Switzerland) by measuring the absorbance at 570 nm.

## 3. Results and Discussion

### 3.1. Characterization

**Scheme 1:**
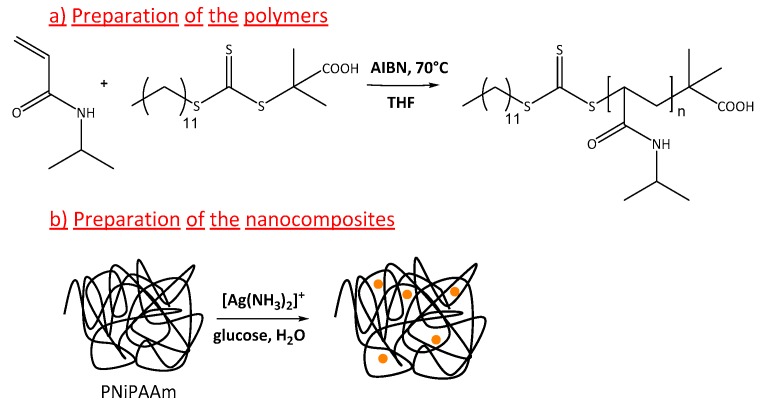
(**a**) Preparation of the polymers by RAFT polymerization; (**b**) Preparation of the nanocomposites.

Four polymers with different molecular weights were synthesized by RAFT polymerization (Scheme 1a). The RAFT polymerization allows a good control over the macromolecular parameters (Table 2). 

**Figure 1:**
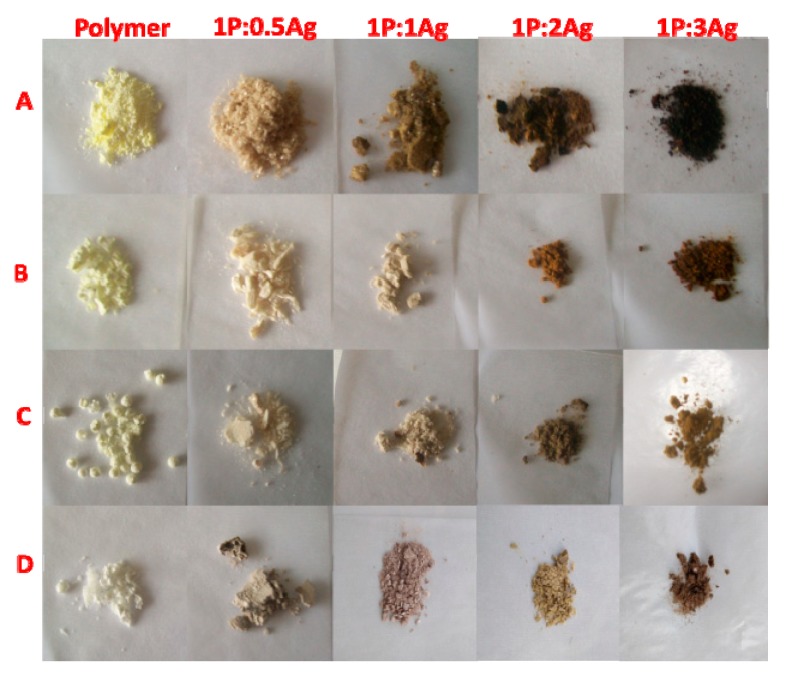
Photographs of the polymers (**A**, **B**, **C**, **D** top to bottom) and the resultant nanocomposites.

For each polymer, four nanocomposites with AgNPs were prepared according to the ratios of polymer:silver [1P:0.5Ag^+^ (**1**), 1P:1Ag^+^ (**2**), 1P:2Ag^+^ (**3**), 1P:3Ag^+^ (**4**)]. Silver nanoparticles (AgNPs) were prepared by in-situ chemical reduction in the presence of the polymers (Scheme 1b). It has already been shown that the polymers stabilize the forming AgNPs by steric repulsion inhibiting the nanoparticles overgrowth [45,46]. Slistan-Grijalva et al. showed that poly(vinylpyrrolidone) (PVP) prevented the particle aggregation during the synthesis and allowed control of the average size [47]. Alcarcon et al. obtained very stable and small AgNPs in the presence of collagen [48]. The polymers also prevent the aggregation of the AgNPs, which is known to reduce the antimicrobial properties [49]. The glucose was chosen as the reducing agent in order to allow a mild reaction. Indeed, too strong agents such as NaBH_4_ or hydrazine lead to a decomposition of the trithiocarbonate group [50]. Our polymers have a specific yellow color (the longer, the lighter the color) due to the trithiocarbonate group. Furthermore, upon the addition of silver, the color turned from yellow to light to dark brown as the silver content increased (Figure 1).

The silver loading was obtained by ICP-OES analyses (Table 3). As expected, a higher silver content is found for smaller polymers. Indeed, the silver loading was based on the molar ratio Polymer:Ag^+^. For each ratio, the silver loading is decreased by half due to the increase of the molecular weight of the polymer.

**Figure 2:**
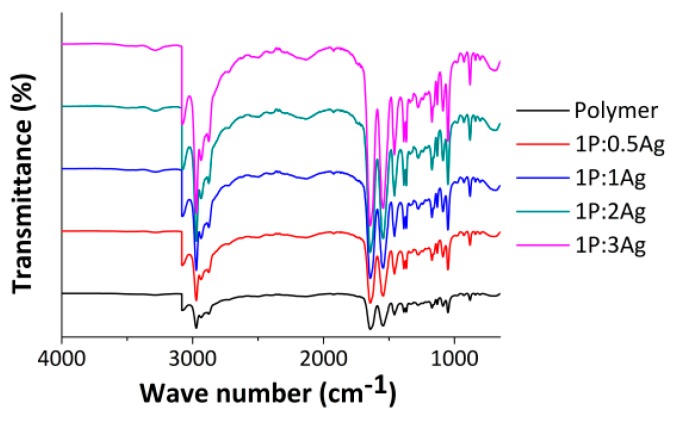
FT-IR spectra of the pure polymer **A** and the nanocomposites at different ratios P:Ag^+^ at RT.

Figure 2 shows the typical FTIR spectra of the TTC-PNiPAAm samples with and without AgNPs. No significant changes were observed in the presence of the AgNPs. The band at 1640 cm^−1^ corresponds to the stretching of the C=O of the amide group. The band at 1538 cm^−1^ is the N–H bending band of the amide [51]. For the polymers **B** to **D**, the spectra can be found in the supporting information (Figures S5 to S7). 

**Figure 3:**
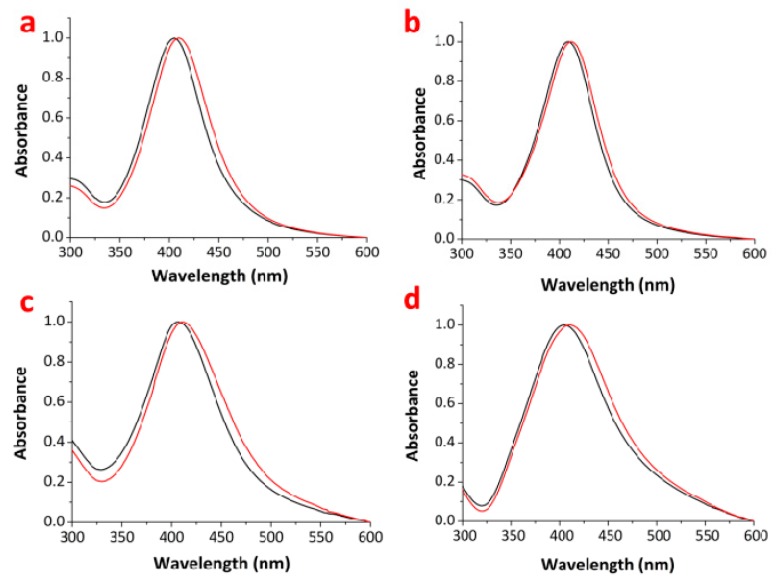
UV-vis spectra of the nanocomposites (**a**) polymer **A**, (**b**) polymer **B**, (**c**) polymer **C**, and (**d**) polymer **D** with the ratio (**3**) at RT (black) and 37 °C (red).

In order to confirm the presence of AgNPs, UV-vis measurements were performed at room temperature on the different samples. An absorption band around 410 nm is observed due to the surface plasmon resonance property, corresponding to AgNPs of ca. 40 nm in diameter [52]. Unfortunately, only the ratios (**3**) (supplementary information, Figure S8) and (**4**) (Figure 3) showed this specific band, while for the other ratios (**1**) and (**2**), the silver percentage was too low for detection.

The UV-vis measurements were also carried out at 37 °C to study the effect of the thermoresponsiveness of the polymer. A shift to a longer wavelength (red shift) was observed at 37 °C for all the polymers. For instance, a shift from 405 to 411 nm was observed for the polymer **A** with a silver ratio (**4**) (Figure 3a). However, the shift does not change with the length of the polymer. This behavior was already observed by Guo et al. [53] where, once heated, their system with PniPAAm showed a peak at 405 nm at 40 °C compared to 397 nm at room temperature (RT). The results suggested that this is mainly due to a modification of the AgNPs dielectric environment. Xu et al. observed similar trends in their experiments. They hypothesized that the shift is due to two phenomena: The shrinkage of the PNiPAAm leads to a more compact conformation diminishing the distance between the NPs and an increase of the refractive index of the polymeric chains [54]. 

**Figure 4:**
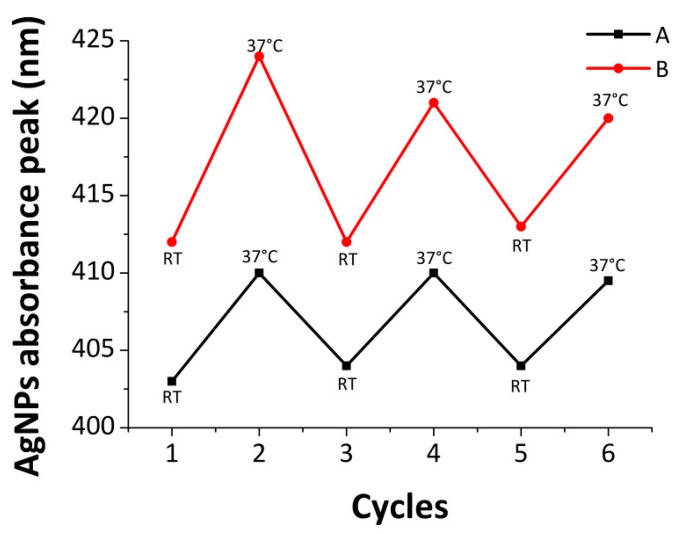
Thermoreversible behavior studied by UV-vis for the polymer **A** with the ratios (**3**) (black) and (**4**) (red).

The temperature-dependent reversibility of the resultant nanocomposites was studied by UV-vis upon three heating/cooling (RT and 37 °C) cycles. It showed that for the polymer **A** with the ratios (**3**) and (**4**) (Figure 4), the maximum absorption of intensity varied according to the temperature and this variation is reversible as the solution is heated or cooled down. The reversible behavior is also confirmed for the other polymers (see supporting information Figures S9–S11). Lin et al. also showed the reversible absorption of their PNiPAAm/Ag for several heating/cooling cycles. They observed, according to the temperature, a variation of the absorption maximum intensity in the range of 0.2 and 1.5 nm. They speculated that upon heating-cooling cycles, their systems showed temperature-dependent reversibility with no aggregation, indicating the good stability of their nanocomposites [55].

**Figure 5:**
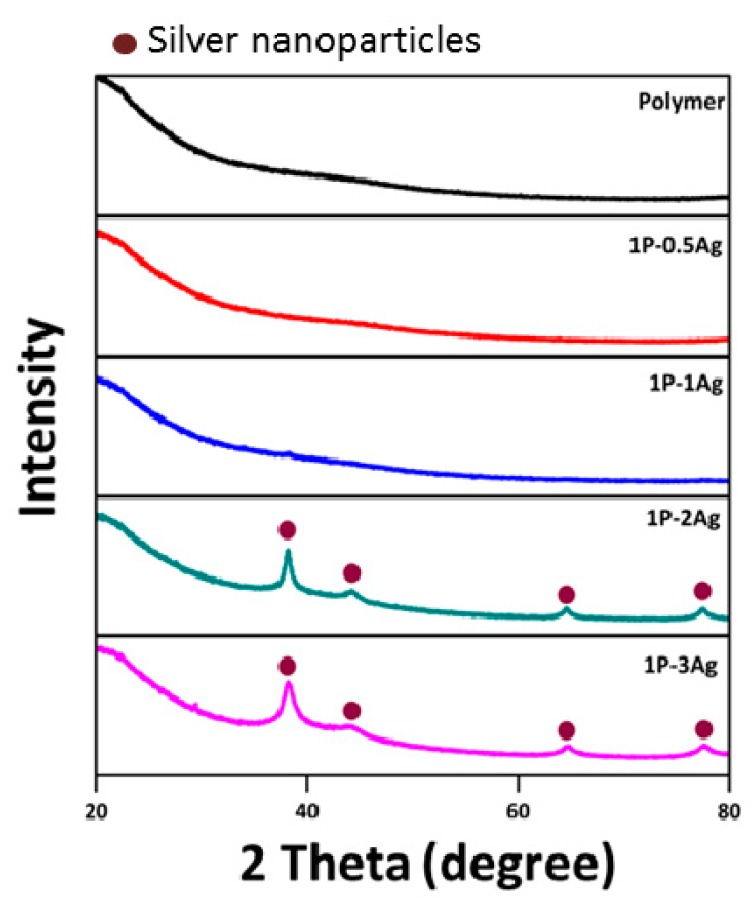
X-ray diffractograms of the polymer **A** and its corresponding nanocomposites at different ratios of silver.

Formation of AgNPs can also be confirmed by X-ray powder diffraction as AgNPs demonstrate a specific pattern [56]. Figure 5 shows the diffractogram of the pure polymer **A** and the resultant nanocomposites. PNiPAAm-TTC showed an amorphous pattern as expected for a non-structured polymer [57,58], but in the presence of silver, characteristic peaks of metallic silver at 38.2°, 44.3°, 64.4°, and 77.7° were observed for the ratios (**3**) and (**4**). These peaks correspond to the (111), (200), (220), and (311) crystal facets of AgNPs [59]. The silver content is too low for detection for the composites with ratios (**1**) and (**2**) (see also Table 4), as typically traces of substances below 3% in a two phases mixture cannot be detected by this method [60]. Additionally, for the other polymers, only the polymer **B** with the ratios (**3**) and (**4**) (supporting information Figure S12) showed the pattern of the AgNPs. The polymers **C** and **D** did not show the specific pattern for AgNPs as the silver loading was too low. 

**Figure 6:**
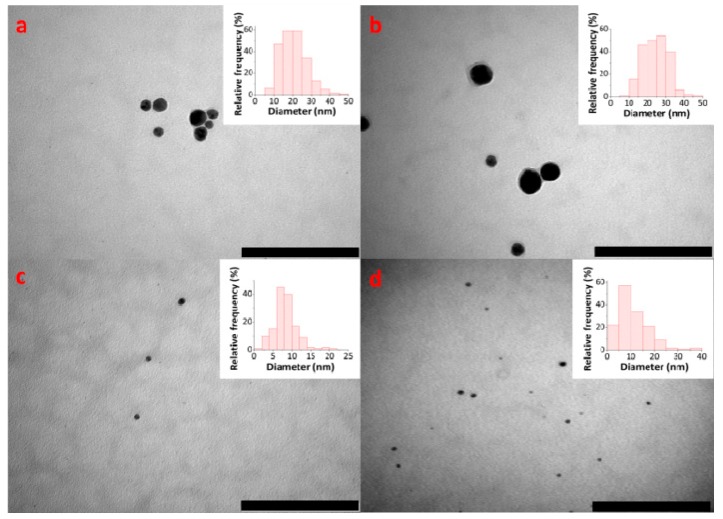
TEM micrographs and size distribution of the AgNPs for the nanocomposites with a ratio (**4**) for Polymer **A** (**a**) (226 NPs), Polymer **B** (**b**) (216 NPs), Polymer **C** (**c**) (143 NPs), and Polymer **D** (**d**) (148 NPs). The scale bar is 200 nm.

TEM analyses were performed to visualize the AgNPs inside the polymer. Quite round-shape particles were observed, independently of the ratio and the molecular weight of the polymer. Figure 6 shows the AgNPs and their distribution for the four polymers at the same ratio (**3**). For polymers **A** and **B** with a lower molecular weight, the silver nanoparticles (AgNPs) have a size between 25 and 30 nm with a broad size distribution and are partially aggregated, while the longer polymers lead to smaller AgNPs < 20 nm with a more narrow size distribution. Therefore, smaller polymers lead to weaker stabilization of the nanoparticles and thus to larger particles [61]. The longer polymeric chains apparently prevent the aggregation of Ag atoms to form large AgNPs [62].

**Figure 7:**
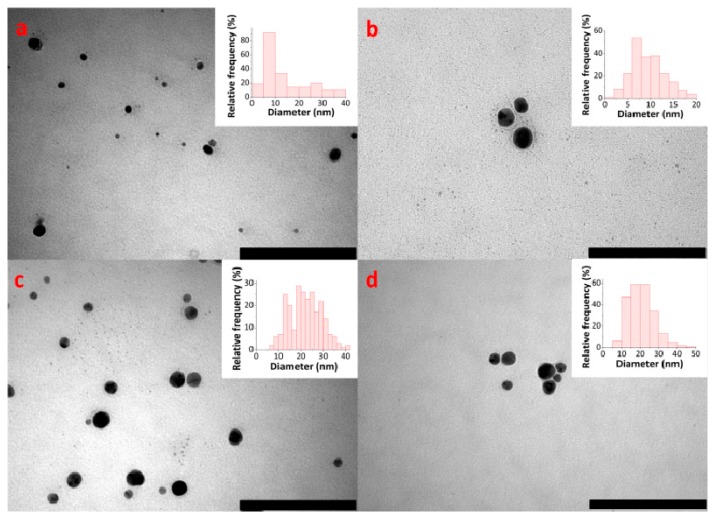
TEM micrographs and distribution of the nanoparticles for the nanocomposites of **A** with the different silver ratios (**1**) (**a**) (208 NPs), (**2**) (**b**) (211 NPs), (**3**) (**c**) (246 NPs), and (**4**) (**d**) (226 NPs). The scale bar is 200 nm.

For the ratios (**3**) and (**4**) (Figure 7), AgNPs have a larger size on average compared to the other ratios due to a decrease of the relative concentration of polymer; less polymeric chains per AgNP are present to stabilize the AgNPs and to prevent their aggregation [63]. This trend is similar for the longer polymers, as larger particles (30 nm) can also be present. However, the polydispersity does not change as a function of the ratios, as for most of the samples, the AgNPs are polydisperse (supporting information Figures S13–S15).

### 3.2. Thermal Properties

**Figure 8:**
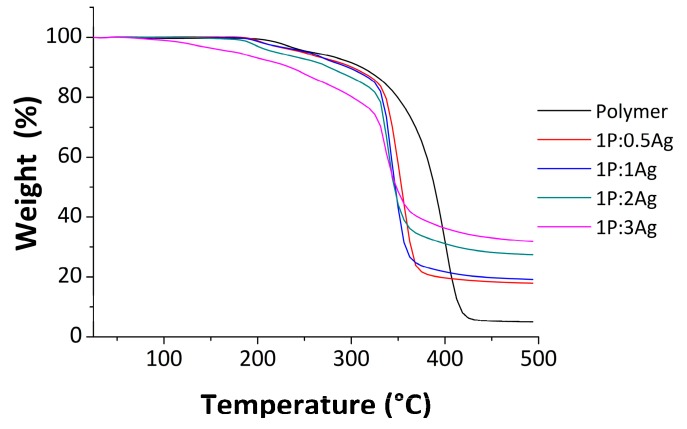
TGA curves of the polymer **A** and the nanocomposites.

In order to determine the influence of silver on the thermal properties in the resultant nanocomposites, thermogravimetric measurements were performed (Figure 8). Two degradation steps were observed: the first one around 200 °C corresponds to the degradation of the trithiocarbonate group, and the second one to the polymer itself at 400 °C [50]. The results demonstrate a lower thermal stability of the nanocomposites at a high silver ratio compared to the pure polymer samples shown in Table 4. The thermal stability thus decreases with the increase of silver content in the composite. This behavior was observed for each polymer (supporting information Figures S16 to S18). These results are consistent with Xu et al. [64] and Lee et al. [65], who hypothesized that the thermic transfer is enhanced due to the homogenous dispersion of silver into the polymeric matrix, leading to a faster degradation [66]. 

**Figure 9:**
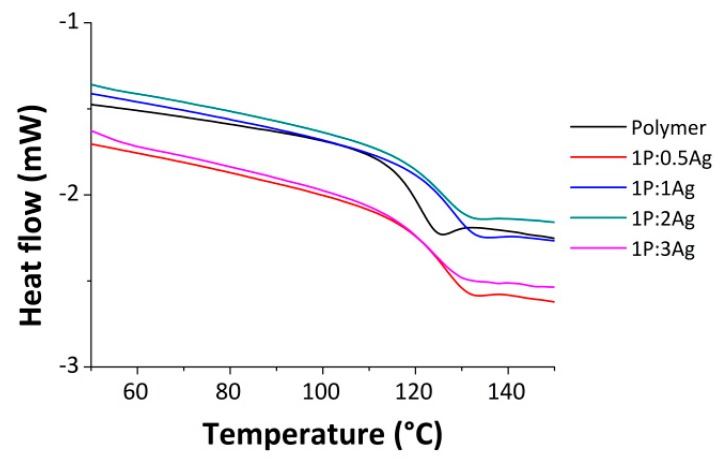
DSC curves of the polymer **A** and the resultant nanocomposites.

DSC measurements were also carried out to study the effect of AgNPs on the glass transition temperature (Figure 9). A slight increase is observed for the ratio (**1**) and (**2**) compared to pure polymer, but the glass transition temperature decreases for higher ratios of silver in the polymers (**3**) and (**4**) (Table 5). The shift to higher temperatures is consistent with the results obtained by Faghihi et al. [67] and Henriquez et al. [68]. These authors speculated that due to the presence of AgNPs, the polymeric chains loose flexibility and freedom of motion, which then enhance the glass transition. Moreover, the strong interaction between the NPs and the polymer by coordination at these ratios might also increase the *T*_g_ [69]. Then, at a higher silver content, the *T*_g_ decreases as the free volume of polymer is affected. The NPs might also weaken the cohesive forces between the polymeric chains, causing the decrease of *T*_g_ [70]. This trend is also observed for the other nanocomposites (supporting information Figures S19–S21).

**Figure 10:**
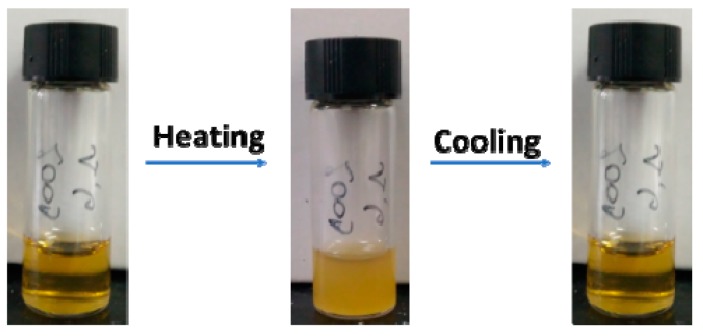
Images of the nanocomposite in H_2_O before and after LCST.

The lower critical solubility temperature was also determined for the polymer and the final nanocomposites. PNiPAAm has a reported LCST in the range of 30–35 °C, but is generally found to be around 32 °C [37] The LCST can be tuned by copolymerization with other monomers, as hydrophilic co-monomers tend to increase it. Furthermore, different parameters could also play a role, such as architectures, end-chains, or more importantly the presence of salts or additives [71]. As AgNPs are present, it is believed to influence the coil-to-globule transition favoring or not favoring the hydrogen bonds [72,73]. Figure 10 represents a random nanocomposite behavior and the LCST reversibility nature. 

The study was performed by measuring the transmittance of the sample solutions in H_2_O at different temperatures (from 32 to 52 °C).

**Figure 11:**
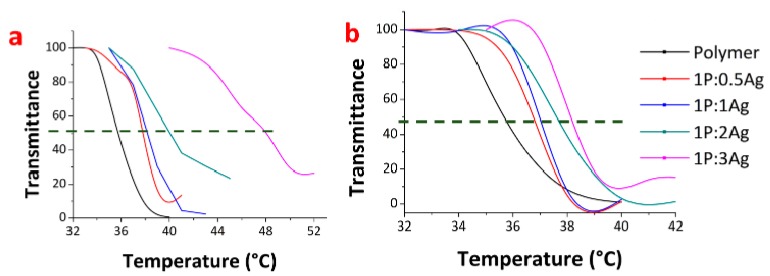
LCST behavior of the polymer **A** (**a**) and **D** (**b**) and the corresponding nanocomposites.

The polymer **A** alone showed an LCST of around 36.1 °C, and as the silver content increases, the cloud point tends to occur at a higher temperature (Figure 11a) (Table 6). The LCST is affected by the end-group, which tends to increase the cloud point (Cp), and Furick et al. already showed that a PNIPAAm without a RAFT agent has an LCST close to 30.5 °C *M*_n_~18,000–20,000 g·mol^−1^) [74]. On the other hand, Qiu et al. observed for the PNiPAAm’s with trithiocarbonate groups, an LCST between 34.1 and 37 °C (*M*_n_~6000–18,000 g·mol^−1^) [75]. For longer polymers such as polymer **D**, the increase in the Cp is less pronounced (Figure 10b), due to the relatively small silver content. The polymers **B** and **C** (supporting information Figure S22) present the same trend in behavior as the other polymers. Therefore, the LCST is modulated by the silver content, and as it increases, the transition phase tends to a higher range of temperatures. The increase is likely due to the coordination of AgNPs to the polymeric chains. Furthermore, the AgNPs can act as chaotropic agents and alter the interactions of the polymeric chains with the solvent, then affecting the transition phase [76,77]. The influence of AgNPs on the LCST has not been investigated yet. The fact that the LCST was increased compared to PNiPAAm itself might be interesting for applications in biological systems, as the average body temperature of animals and humans is in the same range.

### 3.3. Stability of the Nanocomposite

**Figure 12:**
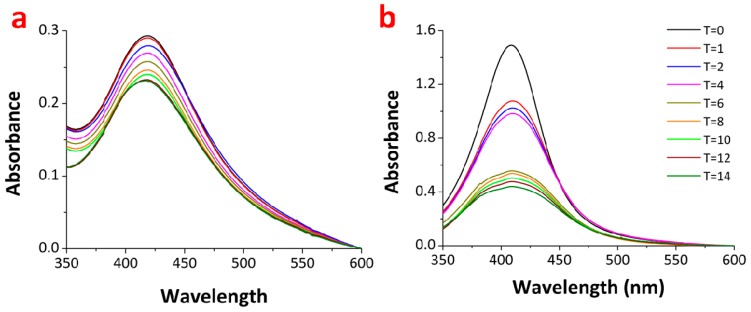
UV-vis spectra of the nanocomposites with the ratio (**4**) for the nanocomposites **A** (**a**) and **B** (**b**) in PBS at 37 °C for two weeks.

In order to test the effect of silver ion release on cells and bacteria, the stability of the nanocomposites with the ratios (**3**) and (**4**) was studied in the phosphate buffered saline (PBS) at 37 °C using UV-vis spectroscopy. PBS was used as a medium to mimic the biological environment. UV-vis spectra were taken after one day, and then every two days over two weeks to visualize the change in the absorbance of the AgNPs. The polymers **C** and **D** could not be studied due to a loss of stability in PBS, as they showed sedimentation after one day at 37 °C. The same phenomenon was also observed in RPMI medium supplemented with 10% serum, but not in water (supporting information Figures S23 and S24). Figure 12 presents the UV-vis spectra for the polymers **A** and **B** with the ratio (**4**). A decrease of the absorbance is observed as the AgNPs dissolve in solution over time to release Ag^+^ [78]. The polymer **A** with ratio (**4**) did not show significant changes in the shape or the breadth of the absorbance band over time. This indicates that the nanocomposite is quite stable in solution and no aggregation occurred. However, the polymer **B** with ratio (**4**) showed a significant decrease of the absorbance after only one day (Figure 12b). After almost one week, the absorption band showed a broader signal, indicating that some aggregation occurred [79]. For the ratio (**3**), the absorption peak did not show significant change (See supporting information Figure S25). In fact, over a prolonged time, it seems that the TTC-PNiPAAms with higher molecular weights are not stable in PBS solution and even tend to precipitate (for Polymers **C** and **D**). This lack of stability was already observed by Lai et al., where their PNiPAAm microspheres tended to aggregate over time in PBS solution, while PNiPAAm-*co*-PAAcs (poly(acrylic acid)) were intact. They speculated that it might be due to an absence of charged groups [80]. In this case, the presence of salts could explain this behavior, as it is known that salts might diminish the solubility of the polymer solution. The salts can disrupt the interactions between the water molecules and the nonpolar groups. It might also affect the volume phase transition, lowering the LCST [81]. Then, due to of the loss of solubility, the polymeric chains tend to aggregate, at the same time lowering the distance between the nanoparticles. 

### 3.4. Silver Release Profile

**Figure 13:**
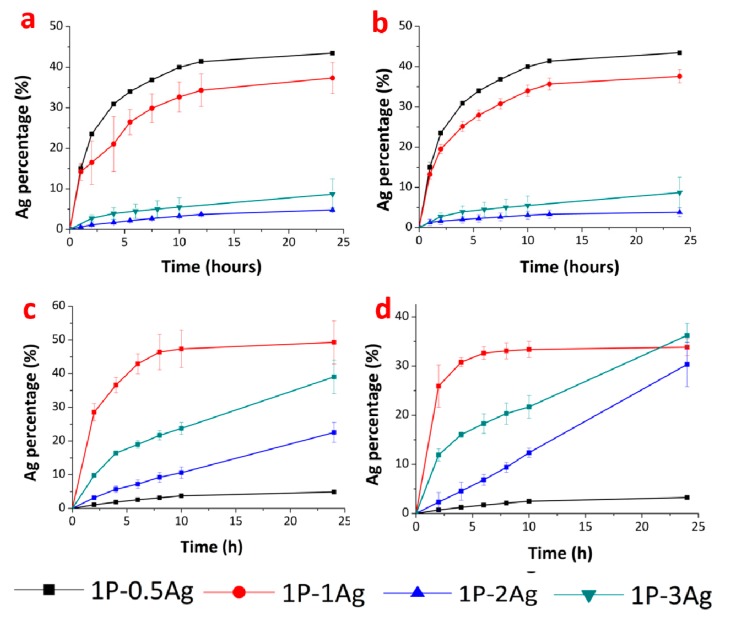
Cumulative silver ion release kinetics at 25 °C (**left**) and 37 °C (**righ**t) for the polymer **A** (**a**,**b**) and for the polymer **D** (**c**,**d**).

All the nanocomposites were investigated for silver ion release in water at room temperature and at 37 °C (Figure 13, supplementary information also Figures S26 and S27). These experiments were done using dialysis tubing, which allows the diffusion of silver ions through the membrane, but not the AgNPs and the polymer. Moreover, this method does not require an additional step to separate the nanoparticles from the sample and allows less contamination of the solution to be analyzed [82]. In all cases, a stable release is achieved after one day, regardless of the polymer and the temperature (Figure 13), and the amount of released silver ions fell below the detection level of ICP. 

For polymer **A**, ca. 40% of the total silver content has been released after 15 h (Figure 13a,b) and the release profiles are independent of the temperature. This shows that the different states of the polymer do not influence the silver ion release, confirming that the AgNPs are not well protected/coordinated by the polymer. 

**Scheme 2:**
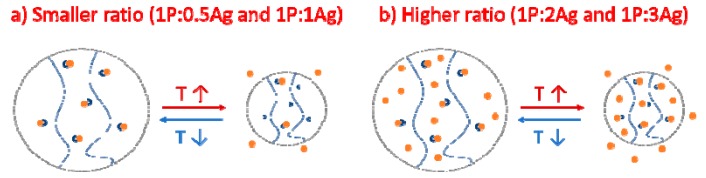
Schematic release of silver at small (**a**) and high (**b**) ratios.

For polymer **D**, the room temperature gives rise to a higher release of silver content of up to ca. 50% in the case of ratio (**2**), while the same composite at a higher temperature releases only ca. 33% of the total silver. However, this behavior was not observed for the ratio (**1**), probably due to the very low silver loading leading to the high entrapment of AgNPs. This trend is reversed when an excess of silver versus polymer was used, as displayed in the green and blue curves in Figure 13c,d. This could in part be explained by the larger amount of TTC-groups able to coordinate to the AgNPs in the ratio (**2**) versus the polymers with a higher silver ratio. Hence, in the collapsed state at a higher temperature, AgNPs are expelled from the polymer more easily and are better dissolved at a higher temperature (Scheme 2).

As an overall trend, shorter polymers (supporting information Figure S26) with the ratios (**1**) and (**2**) released more silver ions than the same polymers with higher silver ratios. This behavior is ratio-dependent and might also be due to the different sizes of the AgNPs. Indeed, smaller nanoparticles will release more due to the higher surface ratio [83]. The ratio (**3**) for the polymer A released less due to the high polydispersity and aggregation of the AgNPs. 

For this ratio and polymer **D**, the temperature can control the release, as the LCST is below 37 °C and hence a phase transition occurs. The polymer present in its collapsed state entraps AgNPs more efficiently with the higher amount to available TTC groups, diminishing the release [84]. 

All nanocomposites except for nanocomposite **D** with ratio (**3**) and (**4**) showed similar trends, with a stabilization after only 24 h with the release of less than 50% of the overall silver amount for ratio (**1**). Such a release profile is typical for sustained and prolonged drug-release behavior characterized by a low release rate [85]. This trend is very interesting for long-term application as the coating should maintain an antimicrobial activity over a long period of time. 

### 3.5. Antimicrobial Properties

#### 3.5.1. *E. coli*

**Figure 14:**
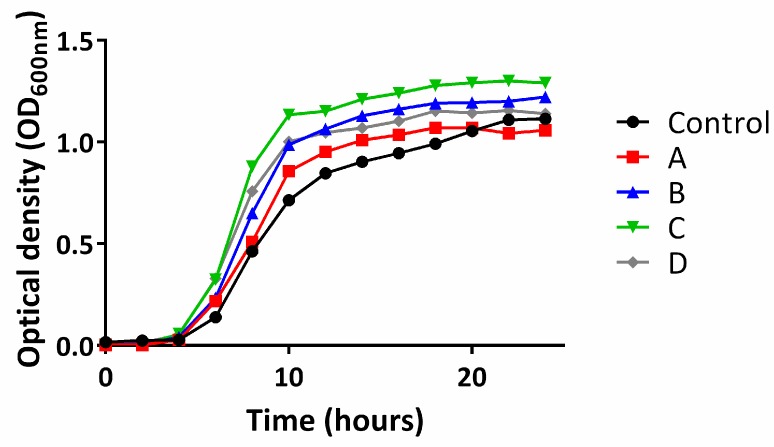
Growth curves of the silver-free polymers at 1 mg/mL against *E. coli* (Control).

In order to assess the antimicrobial activity of these nanocomposites, killing curve assays were achieved in 96-well-plates at 37 °C. These tests were performed using a TECAN reader, analyzing the optical density (OD) at 600 nm for 24 h at different time intervals. First, each silver-free polymer was studied at 1 mg/mL (Figure 14) to check if they exhibited antimicrobial properties. As shown in Figure 14, no inhibition of the bacterial growth was observed, and the OD is evaluated as the same for the bacteria control. 

**Figure 15:**
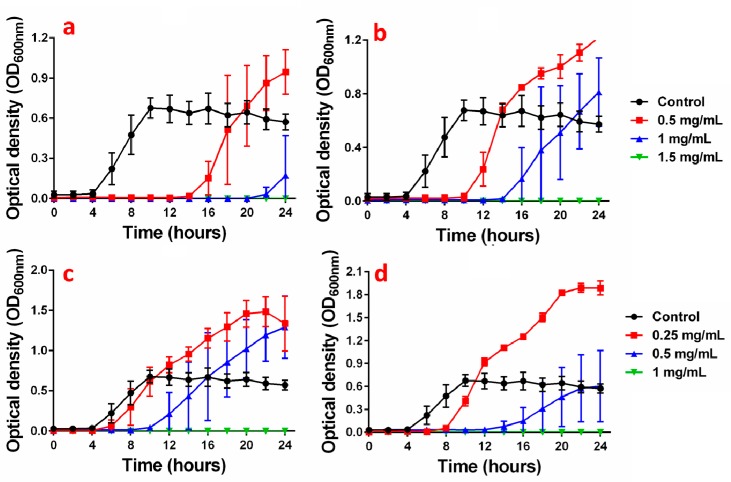
Growth curves of the nanocomposites **A** with the ratios (**1**) (**a**), (**2**) (**b**), (**3**) (**c**), and (**4**) (**d**) at various concentrations against *E. coli*.

Following this, three different concentrations (ranging between 0.5 and 2 mg/mL) were evaluated for each nanocomposite against the *E. coli* strain. This strain is often used as test bacteria due to its easy handling and low associated risk. Figure 15 represents the nanocomposites **A** with each ratio. At small concentrations (<0.5 mg/mL), the OD displayed a higher density compared to the bacteria control, indicating an uncontrolled growth [86]. Higher concentrations showed at least a slight antimicrobial effect or even full killing. The activity is dependent of the ratio and the total silver content. For the smaller ratios (**1**) and (**2**), a higher concentration of 1.5 mg/mL is needed in order to kill the bacteria compared to 1 mg/mL for higher ratios (Figure 15). However, at a concentration of 1 mg/mL, the ratios (**1**) and (**2**) presented an inhibition of bacterial growth for the 12 first hours. The polymer **A** presents the highest antimicrobial activity and bacterial growth inhibition due to the high silver content. The antimicrobial activity could be correlated to the silver release (cumulative in ppb, Supplementary information Figure S28) as the higher ratio (**4**) released more silver than the low ratios (**1**) and (**2**). Indeed, the ratio (**4**) released a larger amount of silver due to higher silver loading as compared to ratio (**1**). 

**Figure 16:**
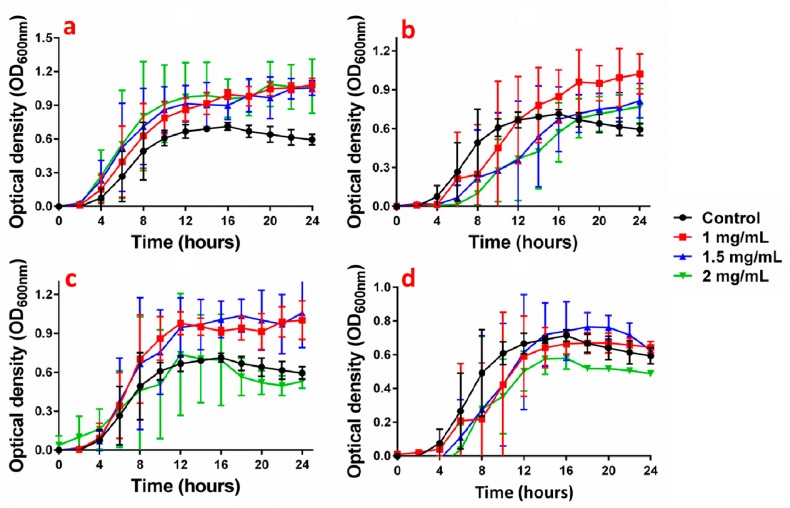
Growth curves of the nanocomposites **D** with the ratios (**1**) Ag (**a**), (**2**) (**b**), (**3**) (**c**), and (**4**) (**d**) at various concentrations against *E. coli*.

The polymer **B** (supporting information Figure S32) showed full killing for the ratios (**1**) and (**2**) at 1.5 mg/mL; however, the ratios (**3**) and (**4**) did not demonstrate bacteria killing at the working concentration range. This fits with the release kinetics experiments, showing that the nanocomposites did not release enough silver within 24 h to kill bacteria. Indeed, the ratios (**3**) and (**4**) displayed a similar release trend (in ppb, supplementary information Figure S29) to the ratios (**1**) and (**2**), and the release is thus not sufficient at 1 mg/mL for the complete killing of bacteria. The polymer **C** (supporting information Figure S33) did not show antimicrobial activity for the smaller ratios (**1**) and (**2**) at the working concentration range. But, for the ratios (**3**) and (**4**), the tests demonstrated bacteria killing from 1.5 mg/mL. The concentration of silver released for these ratios was sufficient to fully kill the bacteria, as shown in the supporting information Figure S30. As observed in the release kinetics analyses, the ratio (**4**) demonstrated the highest release. Figure 16 presents the growth curves for the polymer **D** and the nanocomposites. The nanocomposites did not display bacterial killing after spraying on agar plates but only inhibition at the working concentration range (1–2 mg/mL). At 37 °C, however, as the polymer is in a collapsed state, the release of silver ions is slowed down, as shown in the release experiments (Figure S31). 

The minimum bactericidal concentrations (MBC) were evaluated (Table 7) for a selection of nanocomposites by spreading the sample showing no OD on agar plates after 24 h. The minimum inhibitory concentration (MIC) is defined as the lowest concentration which shows no change in the OD [87].

The MBCs of the polymers **A** and **B** were evaluated at 1.5 mg/mL for the ratio (**1**) and (**2**). For the polymer **B**, a higher concentration is needed for inhibiting bacterial growth for the ratio (**2**) and (**3**) than for the other ratios, even if the silver content is higher. This behavior can be related to the fact that the metal ion release is lower for these ratios and so less silver ions are present to inhibit/kill the bacteria. Also, the AgNPs are bigger in size, leading to a decrease of the antimicrobial effect [88]. The polymer **C** demonstrated full killing at 2 mg/mL for the ratios (**3**) and (**4**), as shown in the release in ppb (Figure S30). Indeed, the amount of silver was sufficient (>200 ppb) to fully kill the bacteria. A similar trend was observed for the polymer **D** (Figure S31). 

#### 3.5.2. *S. aureus*

**Figure 17:**
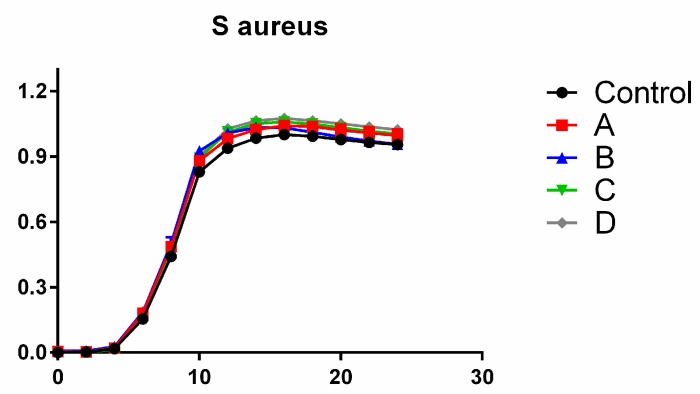
Growth curves of the silver-free polymers at 1 mg/mL against *S. aureus* (Control).

In order to study the activity of the nanocomposites against different bacterial strains, the same antimicrobial tests were performed against Gram positive *S. aureus*. First, each silver-free polymer was studied at 1 mg/mL to check if it exhibited an antimicrobial effect. As shown on the Figure 17, no inhibition of the bacterial growth or killing was observed, suggesting that the polymers themselves did not possess antimicrobial properties. 

**Figure 18:**
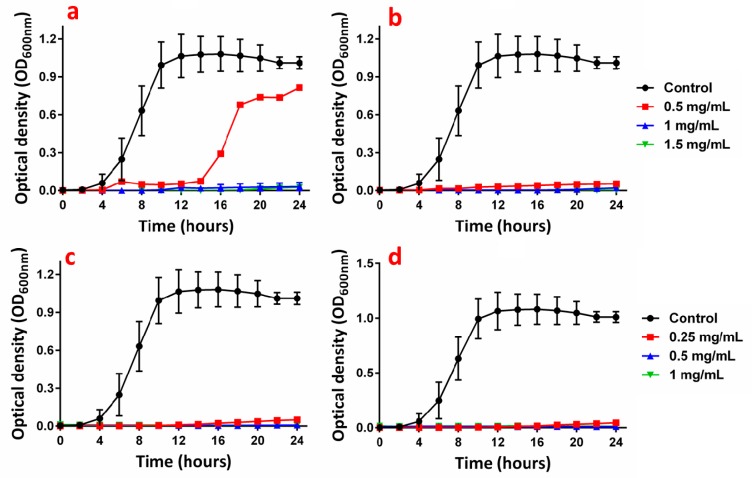
Growth curves of the nanocomposites **A** with the ratios (**1**) (**a**), (**2**) (**b**), (**3**) (**c**), and (**4**) (**d**) at various concentrations against *S. aureus*.

The antimicrobial activity was evaluated at the same concentrations used with *E. coli* (0.25–2 mg/mL depending on the nanocomposites). Figure 18 represents the growth curves for the nanocomposites **A**, and it seems that the nanocomposites severely inhibited the bacterial growth, as attested by a very low OD. However, no killing was observed after spraying on the agar plates for the ratios (**1**) and (**2**) at the working concentration ranges**,** while complete killing was confirmed for the ratios (**3**) and (**4**) at 1 mg/mL. In comparison, studies also showed the same behavior for a PNiPAAm/Ag nanocomposite. The authors also reported a slight inhibition of *S. aureus* growth with a decrease of the logarithmic phase as observed for our materials. However, no killing was induced at the working concentration range [89].

**Figure 19:**
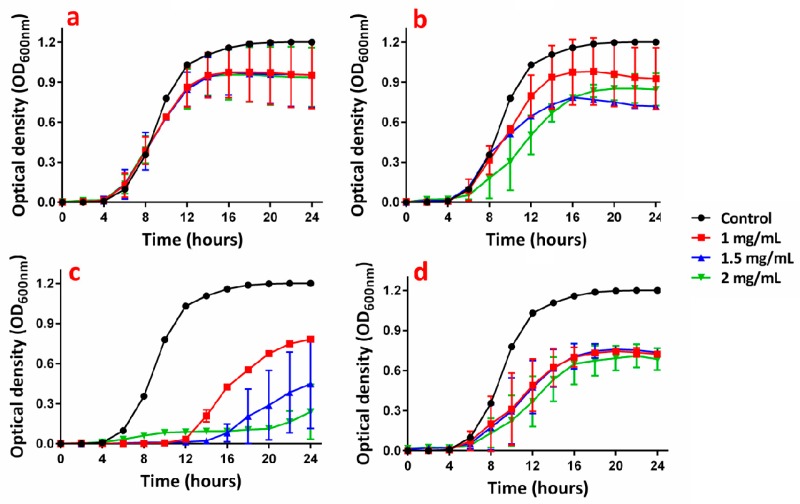
Growth curves of the nanocomposites **D** with the ratios (**1**) (**a**), (**2**) (**b**), (**3**) (**c**), and (**4**) (**d**) at various concentrations against *S. aureus*.

The polymer **B** also demonstrated severe inhibition of bacterial growth (supporting information Figure S34) at the working concentration range. However, it did not demonstrate any bacteria killing after spraying on the agar plate, even for the ratios (**3**) and (**4**), as observed for *E. coli*. However, the polymer **C** (supporting information Figure S35) showed complete bacteria killing for the ratio (**4**) at 2 mg/mL and severe inhibition of the bacterial growth at 1 mg/mL. Figure 19 represents the growth curves of the nanocomposite D. Smaller OD values compared to the control were observed for all the concentrations, suggesting a slight inhibition of the bacterial growth. However, no killing was demonstrated, as observed for the ratio (**4**) against *E. coli*. 

As for *E. coli*, the MBC and MIC were evaluated against *S. aureus* (Table 8). Even if the nanocomposite materials severely inhibited the bacteria growth, almost no killing was observed at the working concentration range. In comparison, the nanocomposites demonstrated a better antimicrobial effect against *E. coli* for the same concentrations. The Gram positive bacteria are in general more tolerant to silver than Gram negative bacteria [90]. This behavior can be easily explained by the cell wall structure differences. Indeed, *S. aureus*, Gram positive bacteria, possess multiple layers of peptidoglycan in comparison to Gram negative bacteria, which present a thin peptidoglycan layer. Therefore, a higher concentration is needed to induce structural damages to the membranes of *S. aureus* than for *E. coli* [91]. Moreover, due to this thicker layer, the Gram positive bacteria allow less silver to penetrate the cell and to reach the cytoplasmic membrane [92].

### 3.6. Cell Viability

**Figure 20:**
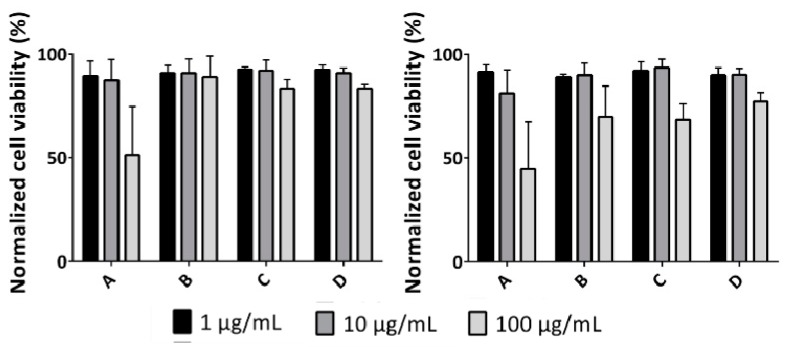
Cell viability of L-929 fibroblasts exposed to different concentrations (1, 10, and 100 µg/mL) of polymers **A**, **B**, **C**, and **D** for 24 h (**left**) and 72 h (**right**) with the ratio (**3**). The effect of polymers was determined by an MTT assay. Results are expressed relative to the untreated control, which was defined as 100%. Mean ± standard deviation (SD) values from at least three independent experiments are shown.

The effect of silver content on the proliferation and metabolic status of L-929 fibroblasts was investigated by an MTT assay following 24 and 72 h (Figure 20).

After 24 h (Figure 20, left), the viability of cells exposed to the tested concentration range (1, 10, and 100 µg/mL) of polymers **A**, **B**, **C**, and **D** with increasing molecular weight and decreasing silver content, remained between 83–92% in comparison to untreated control cells (representing 100% cell viability), and thus displayed good biocompatibility. At this time point, fibroblasts were the most sensitive when treated with the highest dose (100 µg/mL) of polymer **A** having the highest silver content (6.53 Ag w%) that resulted in decreasing the cell viability to 51%. A similar trend was observed at a longer exposure time, after 72 h (Figure 20, right), with respect to lower doses of polymers (1 and 10 µg/mL), resulting in high cell viability (80–90%) compared to the untreated control. After a longer incubation period, the cytotoxic effect was more prominent at 100 µg/mL, where cells exposed to polymer **A** exhibited the lowest viability, similar to a shorter incubation time (45% live cells), while polymer **D** was the least toxic (with 77% live cells).

The cytoxicity is linked to the silver loading, as the cells are more susceptible to be killed. The polymer **A** shows the highest cytotoxicity due to this high content. Based on the total Ag concentration, which is superior to 25 µg/mL, cytoxicity could be induced for most of nanocomposites **A** and nanocomposite **B** with the ratio (**4**). This concentration was shown to be the maximal concentration which still displays biocompatibility against L-929 fibroblasts in-vitro [93]. 

At this point, the AgNPs did not play a role in the toxicity as the polymer **A** presents the biggest AgNPs. It is believed that the smaller NPs have a higher toxicity as they can be potentially more easily distributed into the cells [94]. However, at this point, the aggregation of AgNPs as seen for the polymer **A** can also contribute to the toxicity. Indeed, it has been shown that aggregation may increase the toxicity [95]. 

## 4. Conclusions

We reported the preparation of thermoresponsive nanocomposites based on TTC-PNiPAAm and AgNPs in a reproducible way. Polymers with a low molecular weight and thus short polymer strands lead to larger AgNPs as compared to polymers with a higher molecular weight, due to a worse stabilization and steric effect. Incorporation of the silver changes the thermal properties and also the internal motion of polymeric chains. The presence of silver was shown to decrease the thermal stability of the polymer, leading to a faster thermic degradation. Also, the presence of silver affects the phase transition of the polymer and tends to increase the LCST. The cumulative release of silver ions did not show temperature dependence expect for the polymer **D** with the ratio (**2**) due to a low LCST. Due to the collapsing of the polymeric chains, the release was a bit decreased at 37 °C as compared to room temperature release. The materials demonstrated a typical sustained release profile, making them suitable for long-term applications. More importantly, the nanocomposites demonstrated slight or strong antimicrobial properties depending on the silver loading. The nanocomposites demonstrated a bactericidal effect for *E. coli*, while for *S. aureus*, the bacteriostatic effect is more pronounced. These materials thus have a high potential as coatings for biomaterials.

## Figures and Tables

**Table 1:** Ratio targeted between TTC-PNiPAAm and silver, and abbreviations.

Targeted Ratio TTC-PNiPAAm:Ag^+^	Abbreviation
1P:0.5Ag^+^	1
1P:1Ag^+^	2
1P:2Ag^+^	3
1P:3Ag^+^	4

**Table 2:** General parameters of the synthesized TTC-PNiPAAm samples.

Label	Sample	DP Targeted	DP Obtained	Conv (%) ^a^	*M*_n_ NMR (g.mol^−1^) ^b^	Đ_M_^c^
**A**	TTC-PNiPAAm 25	25	24	94	3080	1.1
**B**	TTC-PNiPAAm 50	50	49	95	5350	1.1
**C**	TTC-PNiPAAm 100	100	103	99	12,020	1.02
**D**	TTC-PNiPAAm 200	200	167	96	18,470	1.2

^a^ Obtained by ^1^H-NMR spectroscopy, ^b^ Determined by ^1^H-NMR spectroscopy, ^c^ Determined by GPC analyses in THF.

**Table 3:** Silver loading (%) of the different nanocomposites.

Nanocomposite	1P:0.5Ag^+^ (1)	1P:1Ag^+^ (2)	1P:2Ag^+^ (3)	1P:3Ag^+^ (4)
**A**	1.53	3.24	6.53	10.37
**B**	0.44	1.65	3.65	4.69
**C**	0.42	0.83	1.74	1.93
**D**	0.29	0.53	1.03	1.62

**Table 4:** Degradation temperatures of the samples.

Nanocomposite	Degradation Temperature (°C)				
	Polymer	1P:0.5Ag^+^ (1)	1P:1Ag^+^ (2)	1P:2Ag^+^ (3)	1P:3Ag^+^ (4)
**A**	400	350	340	335	330
**B**	400	360	355	340	335
**C**	400	380	365	355	345
**D**	400	390	375	365	350

**Table 5:** Glass transition temperatures of the different samples.

Nanocomposite	Glass Transition Temperature (°C)				
	Polymer	1P:0.5Ag^+^ (1)	1P:1Ag^+^ (2)	1P:2Ag^+^ (3)	1P:3Ag^+^ (4)
**A**	119	124	126	126	126
**B**	128	129	130	127	127
**C**	133	135	135	134	131
**D**	133	135	135	123	129

**Table 6:** Lower critical solubility temperatures of the polymers and the final composites.

Nanocomposite	Lower Critical Solubility Temperature (°C)				
	Polymer	1P:0.5Ag^+^ (1)	1P:1Ag^+^ (2)	1P:2Ag^+^ (3)	1P:3Ag^+^ (4)
**A**	36.1	37.7	38.1	39.2	48.5
**B**	35.5	38.5	39.4	42.3	42.6
**C**	35.4	38	38.2	40.5	41.1
**D**	35.6	36.5	36.8	37.5	38.1

**Table 7:** MBC (left) and MIC (right) of the nanocomposites in mg/mL against *E. coli*.

Nanocomposite	1P:0.5Ag^+^ (1)		1P:1Ag^+^ (2)		1P:2Ag^+^ (3)		1P:3Ag^+^ (4)	
**A**	1.5	>1	1.5	>1	1	>0.5	1	>0.5
**B**	1.5	>1	1.5	>1	1	>0.5	1	>0.5
**C**	>2	>2	>2	>2	2	1.5	2	1.5
**D**	>2	>2	>2	>2	>2	2	>2	2

**Table 8:** MBC (left) and MIC (right) of the nanocomposites in mg/mL against *S. aureus*.

	1P:0.5Ag^+^ (1)		1P:1Ag^+^ (2)		1P:2Ag^+^ (3)		1P:3Ag^+^ (4)	
**A**	>2	1	>2	1	1	0.5	1	0.5
**B**	>2	>1.5	>2	>1.5	>1.5	>1	>1.5	>1
**C**	>2	2	>2	2	2	1.5	2	1.5
**D**	>2	>2	>2	>2	>2	>2	>2	>2

## Supplementary Materials

The following are available online at http://www.mdpi.com/2073-4360/10/6/665/s1.
