# Supplementary material for: Trithiocarbonate-Functionalized PNiPAAm-Based Nanocomposites for Antimicrobial Properties
Source: Polymers (Basel). 2018 Jun 14;10(6):665. doi: 10.3390/polym10060665 (PMC6404129; doi:10.3390/polym10060665)
Supplement: Supplementary file 1 [file polymers-10-00665-s001.pdf]

Supplementary information for

Trithiocarbonate-functionalized PNiPAAm-based nanocomposites for antimicrobial properties

Milène Tan[a], Lenke Horvàth[a], Priscilla S. Brunetto[a], Katharina M. Fromm[a*]

[a]University of Fribourg, Department of Chemistry, Chemin du Musée, 9. 1700 Fribourg, Switzerland.

E-mail: Katharina.fromm@unifr.ch

Table of contents

1\ Structural characterization of the polymers and the nanocomposites

1.1 ^{1}H-NMR-spectra of the poly(N-isopropylacrylamide)'s……………………………………………………….S1-4

1.2 FT-IR spectra of the nanocomposites B, C and D……………………………………………………………..S5-7

1.3 UV-Vis spectra of the nanocomposites with the ratio (**3**)……………………………………………………S8

1.4 Thermoreversible behavior studied by UV-vis upon heating-cooling cycles…………………………S9-11

1.4 X-ray diffractogram of the polymer **B** and the nanocomposites………………………………………..S12

1.5 TEM micrographs of the polymer B, C, D and the nanocomposites…………………………………….S13-15

2\ Thermal properties

2.1 TGA curves of the polymers **B**, **C** and **D** and the nanocomposites………………………………………S16-18

2.2 DSC traces of the polymers **B**, **C** and **D** and the nanocomposites………………………………………S19-21

2.3 LCST behaviors of the polymers **B** and **C** and the nanocomposites…………………………………………S22

3\ Stability studies

3.1 For all the nanocomposites the ratio (**3**) and (**4**) inH_2O…………………………………………………..S23-24

3.2 For the polymers **B** and **C** with the ratio (**3**) in PBS…………………………………………………………….S25

4\ Release kinetics

4.1. Silver release profile of nanocomposite B and C……………………………………………………………...S26-27

4.2. Silver release profile of nanocomposite A, B, C and D……………………………………………………...S28-31

5\ Antimicrobial properties for the polymers

5.1. Killing curves of the nanocomposite B and C with *E. coli* …………………………………………………S32-33

5.2. Killing curves of the nanocomposite B and C with S. aureus………………………………………………S34-35

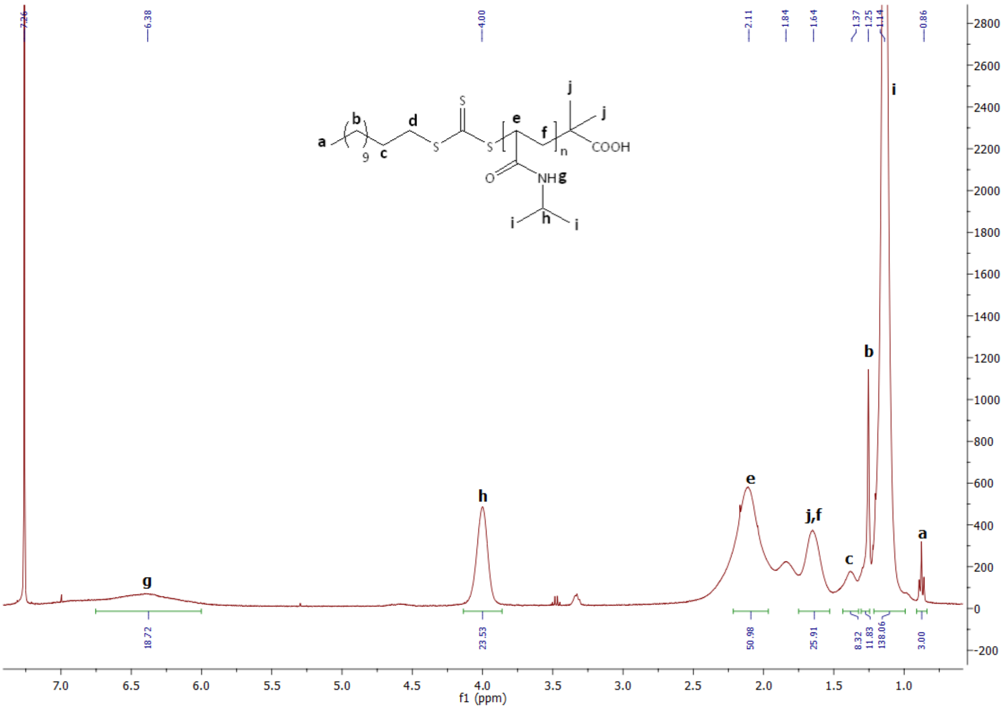

Fig. S1 ^{1}H-NMR (400 MHz) spectrum of the polymer **A**

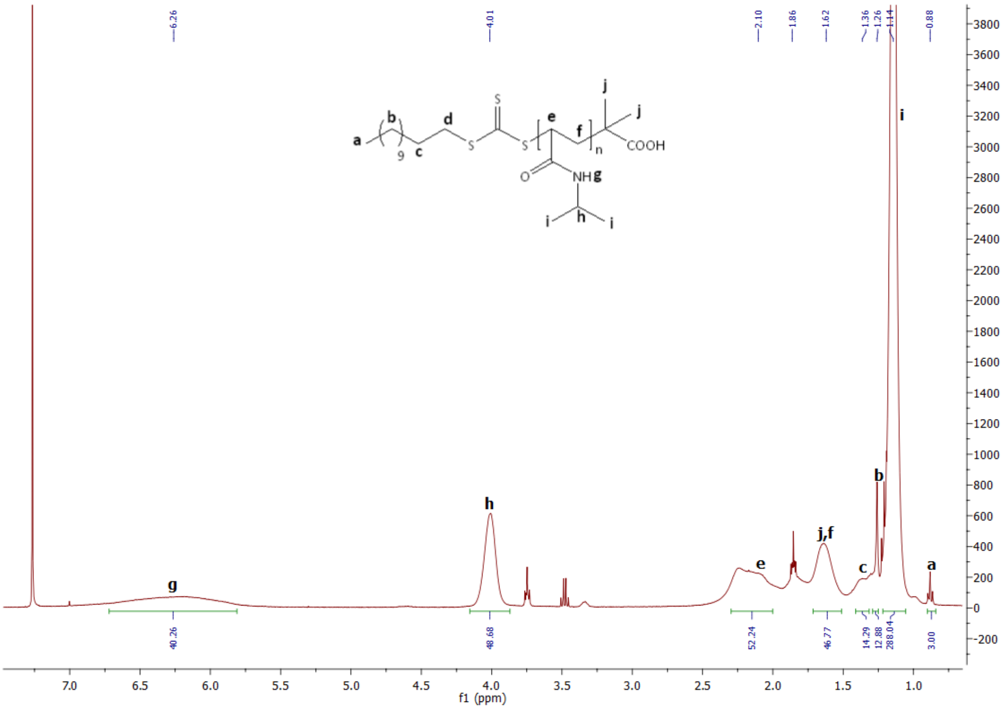

Fig. S2 ^{1}H-NMR (400 MHz) spectrum of the polymer **B**

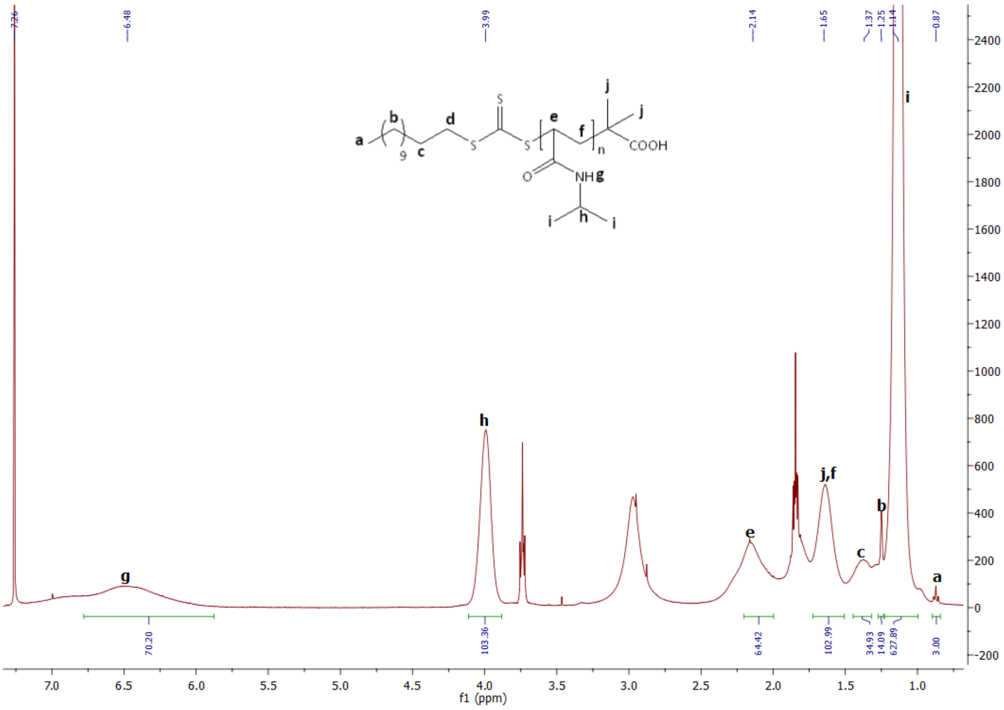

Fig. S3 ^{1}H-NMR (400 MHz) spectrum of the polymer **C**

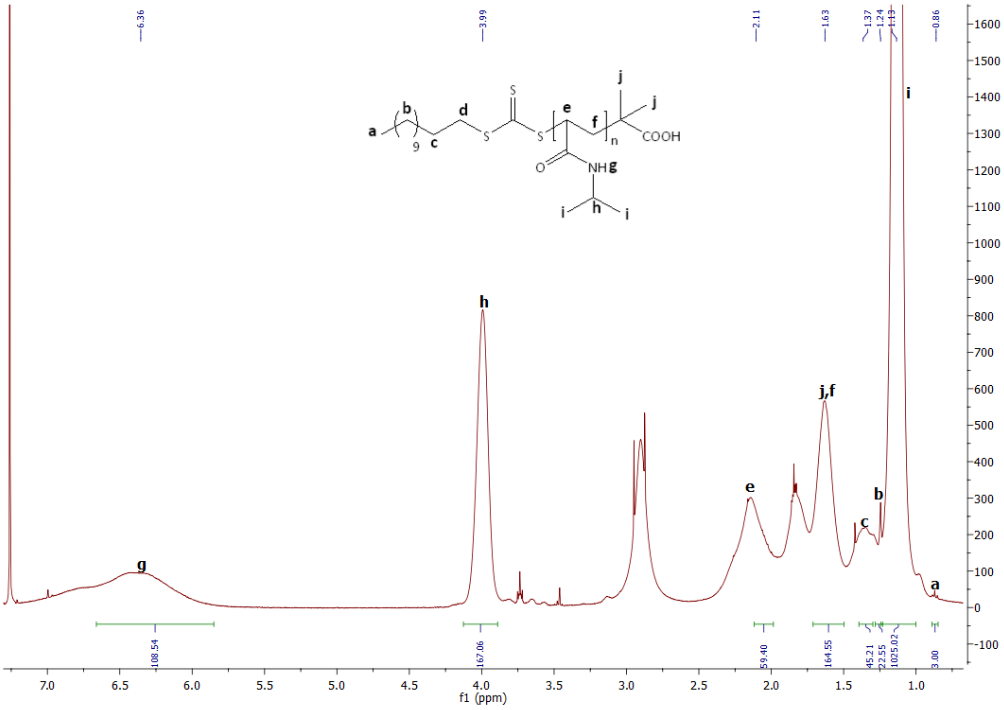

Fig. S4 ^{1}H-NMR (400 MHz) spectrum of the polymer **D**

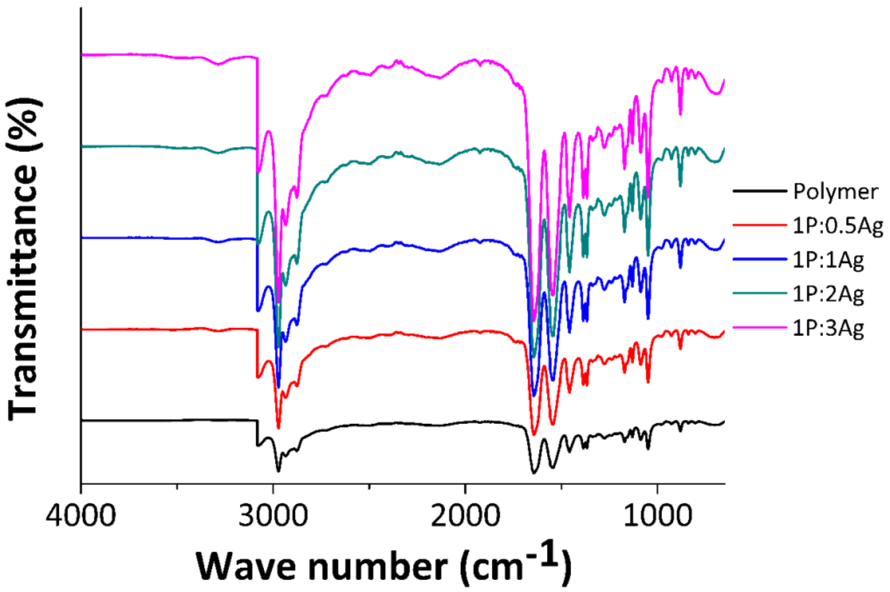

Fig. S5: FT-IR spectra of the polymer **B** and the nancomposites

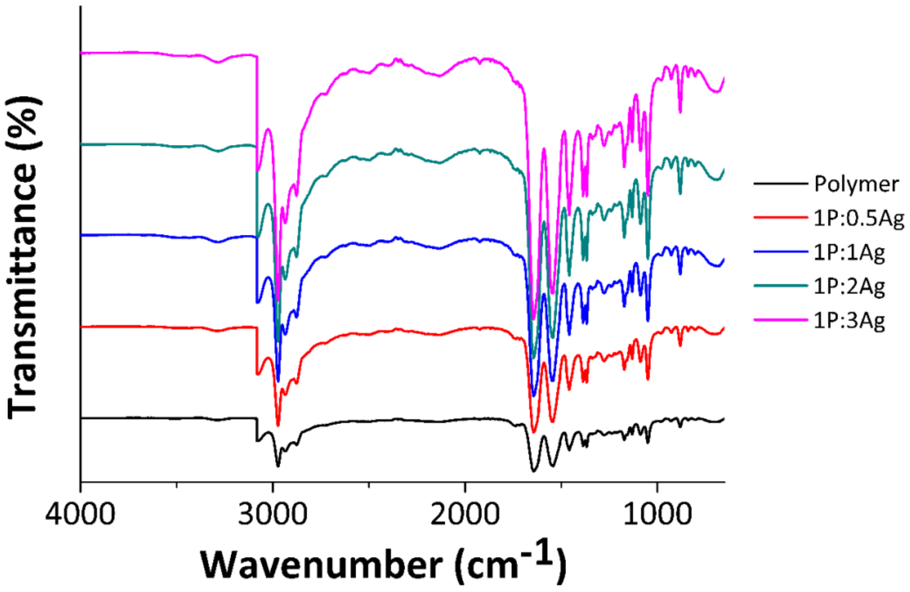

Fig. S6 : FT-IR spectra of the polymer **C** and the nancomposites

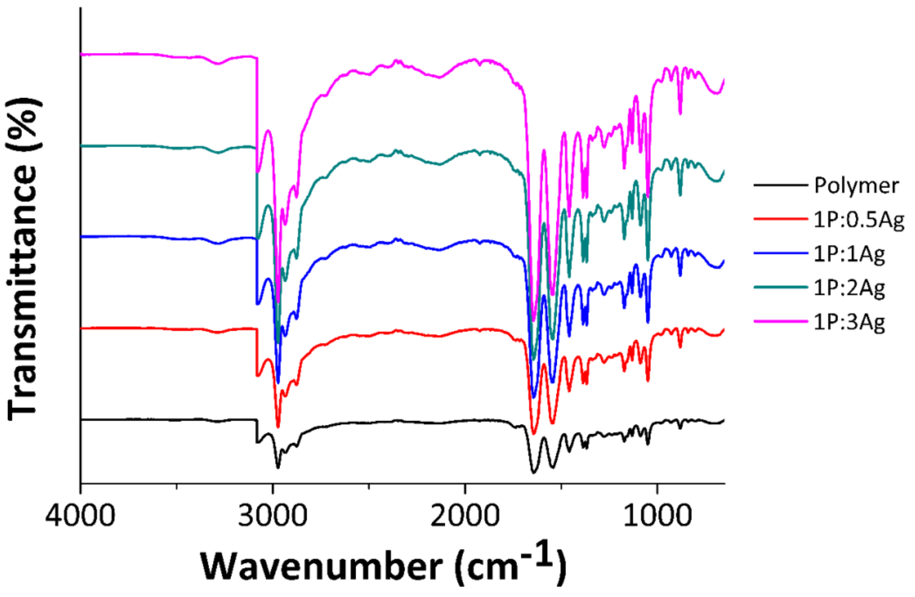

Fig. S7 : FT-IR spectra of the polymer **D** and the nancomposites

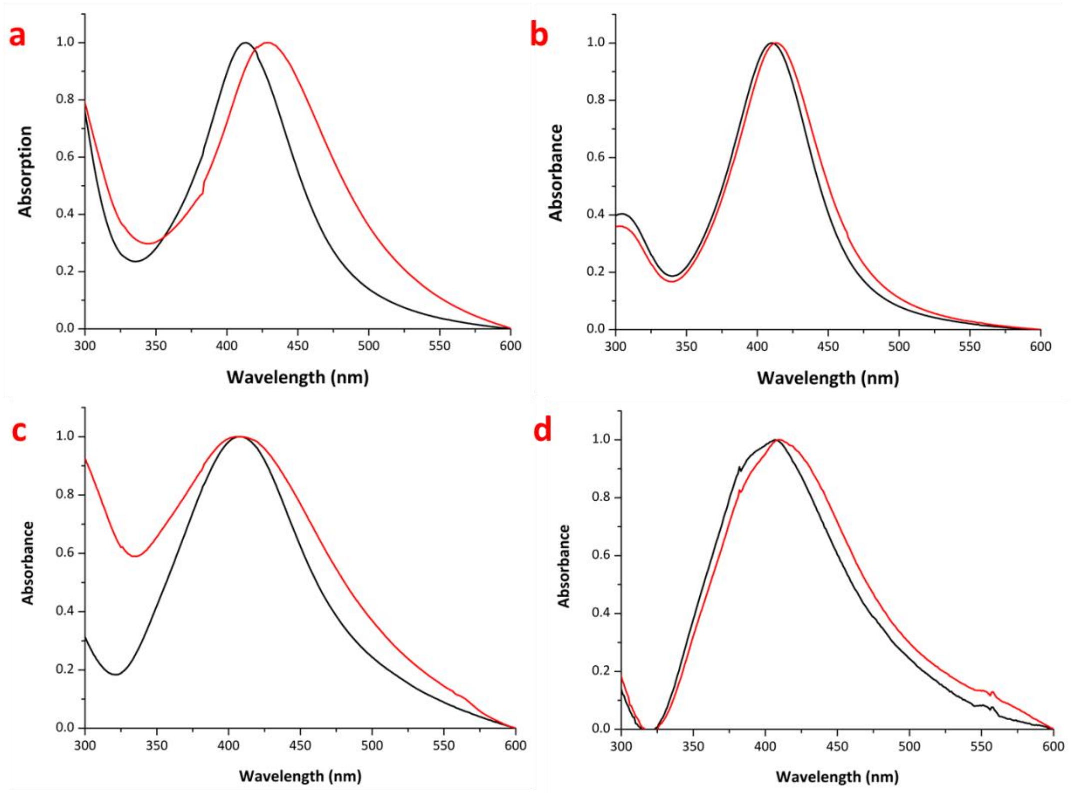

Fig. S8: UV-vis spectra of the nanocomposites a) polymer **A**, b) polymer **B**, c) polymer **C**, d) polymer **D** with the ratio **(3)** at RT (black) and 37 °C (red)

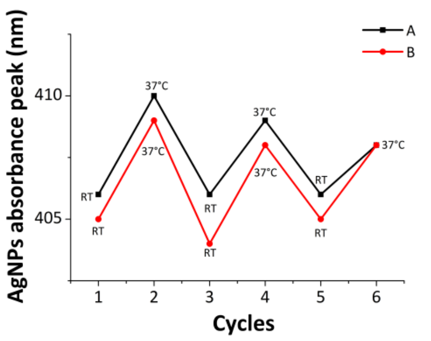

Fig. S9 Thermoreversible behavior studied by UV-vis for the polymer **B** with the ratios **(3)** (A) and **(4)** (B)

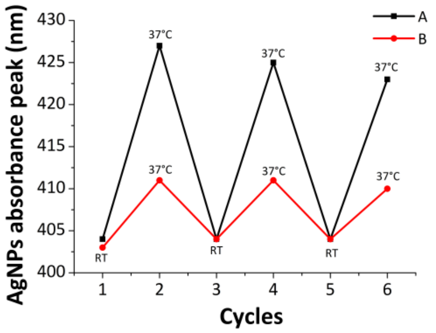

Fig. S10 Thermoreversible behavior studied by UV-vis for the polymer **C** with the ratios **(3)** (A) and **(4)** (B)

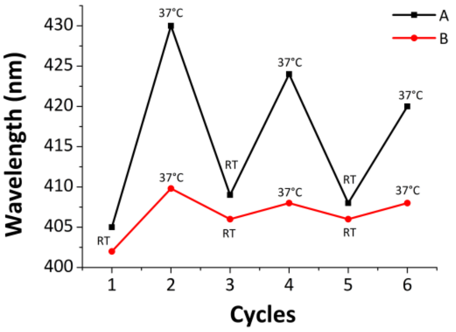

Fig. S11 Thermoreversible behavior studied by UV-vis for the polymer **C** with the ratios **(3)** (A) and **(4)** (B)

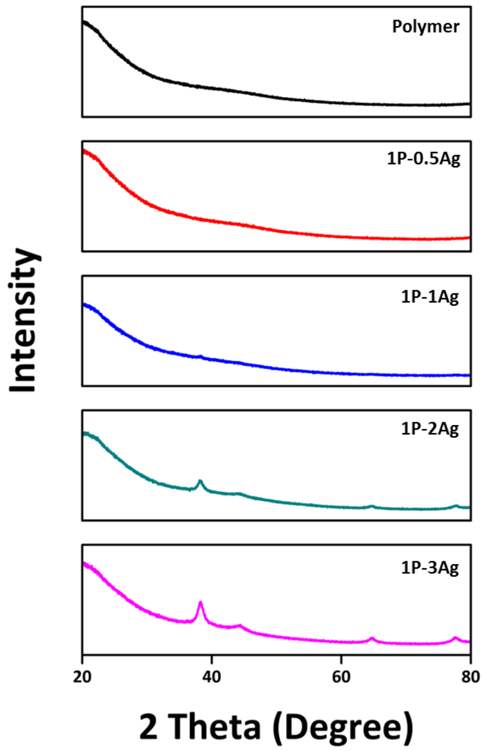

Fig. S12 X-ray diffractogram of the polymer **B** and the nanocomposites

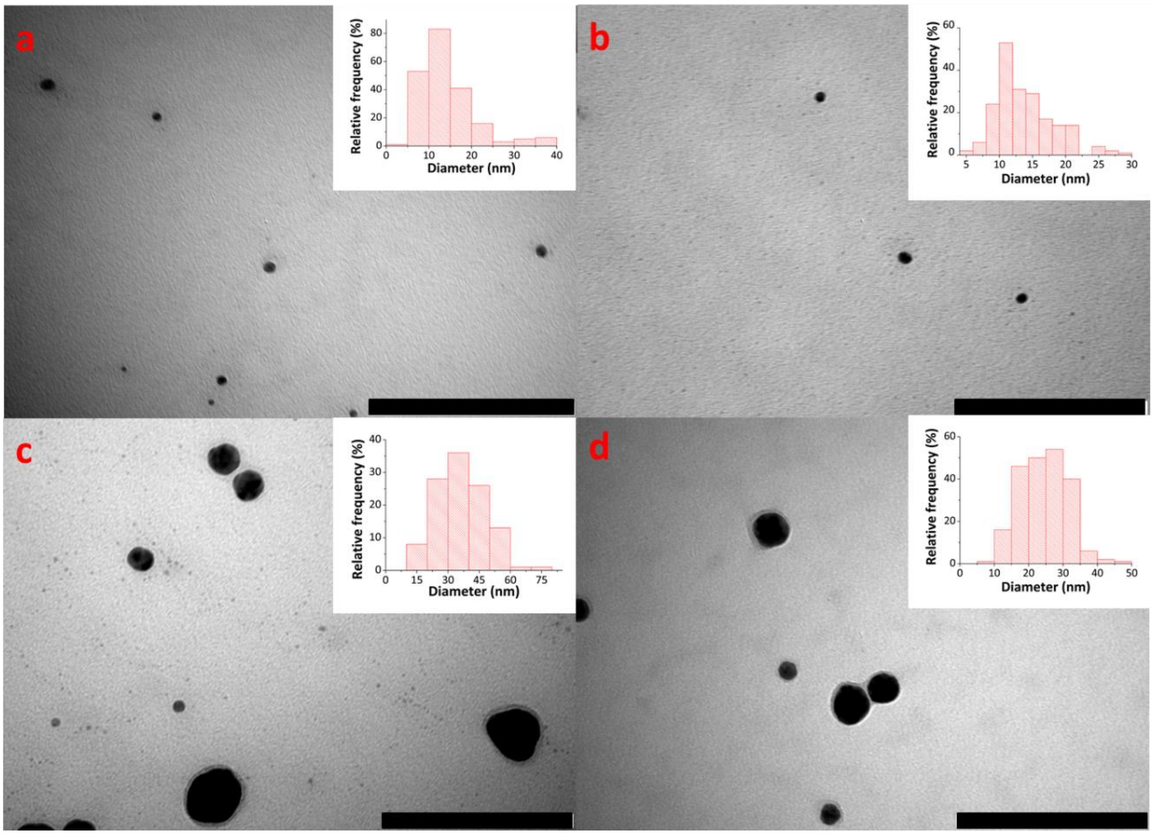

Fig. S13 TEM micrographs and distribution of the nanoparticles for the nanocomposites **B** with the different ratios (**1**) (a) (203 NPs), (**2**) (b) (208 NPs), (**3**)(c) (113 NPs), (**4**) (d) (216 NPs).

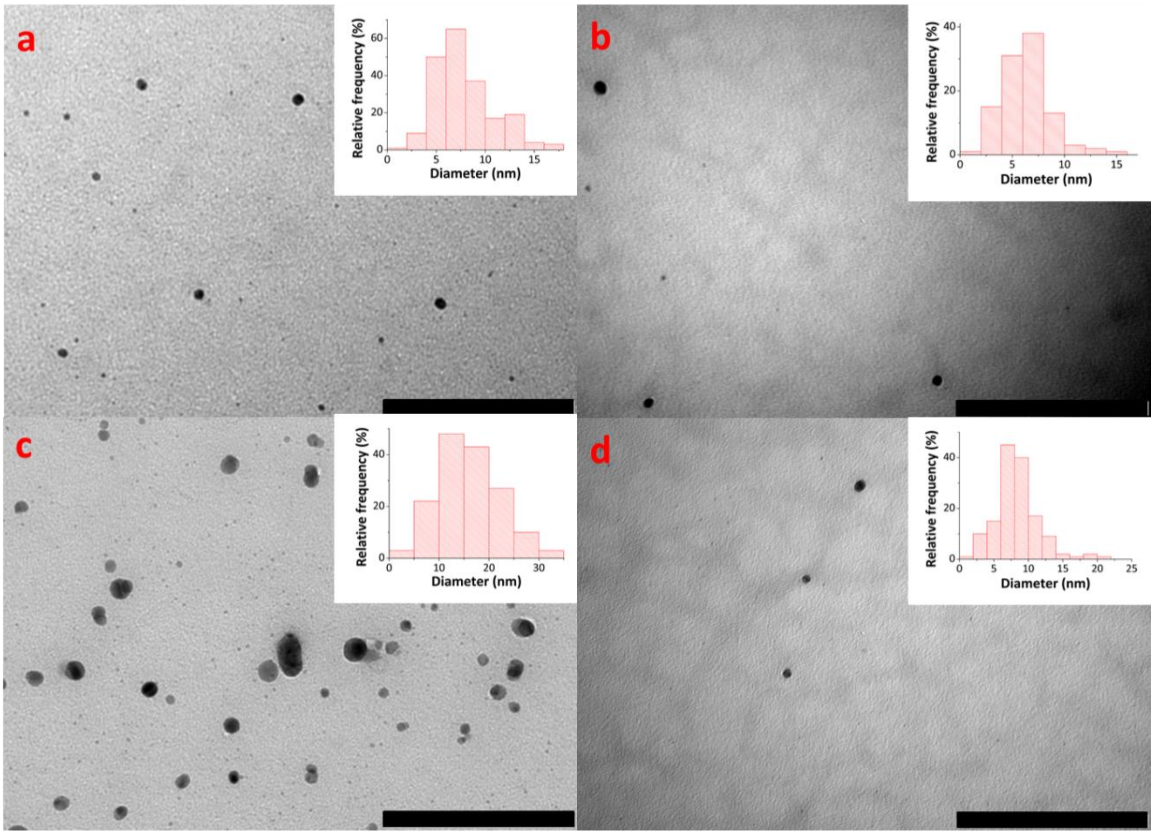

Fig. S14 TEM micrographs and distribution of the nanoparticles for the nanocomposites **C** with the different ratios (**1**) (a) (205 NPs), (**2**) (b) (105 NPs), (**3**)(c) (156 NPs), (**4**) (d) (143 NPs)

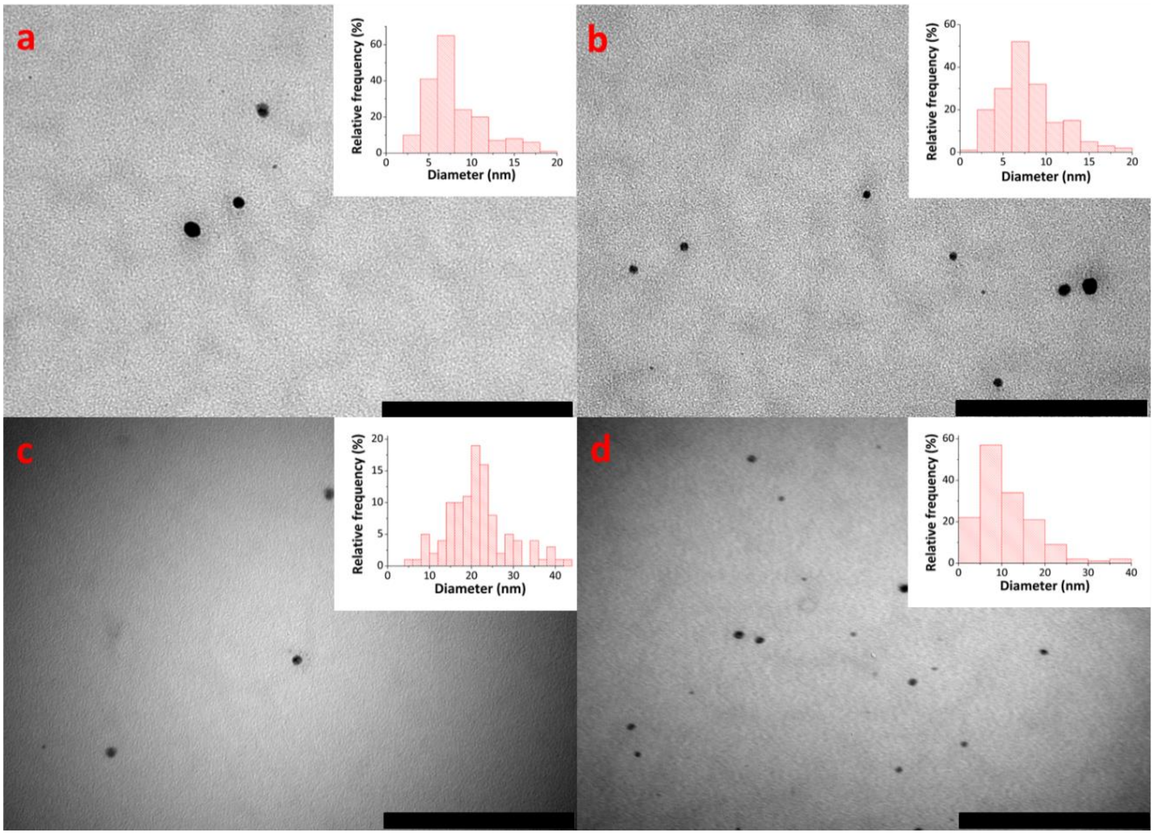

Fig. S15 TEM micrographs and distribution of the nanoparticles for the nanocomposites **D** with the different ratios (**1**) (a) (182 NPs), (**2**) (b) (174 NPs), (**3**)(c) (108 NPs), (**4**) (d) (148 NPs).

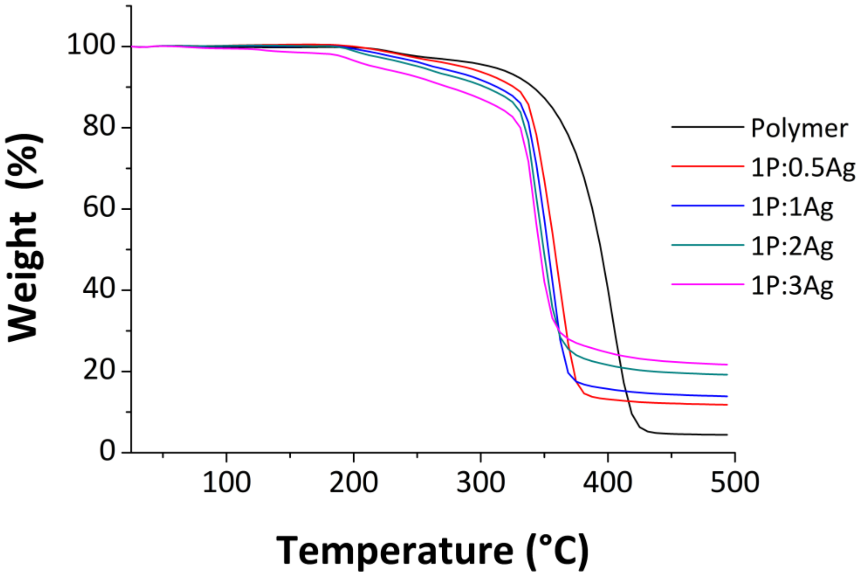

Fig. S16 TGA traces of the polymer **B** and the nanocomposites

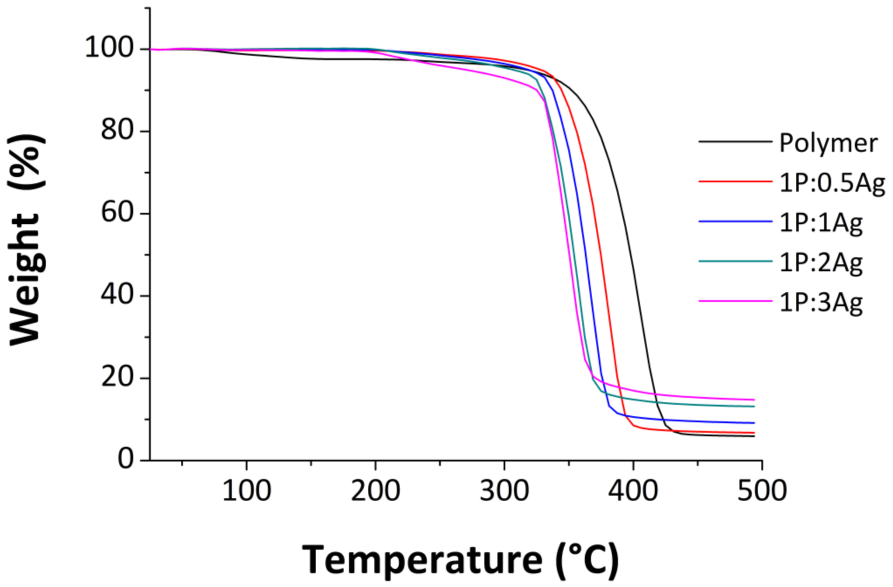

Fig. S17 TGA traces of the polymer **C** and the nanocomposites

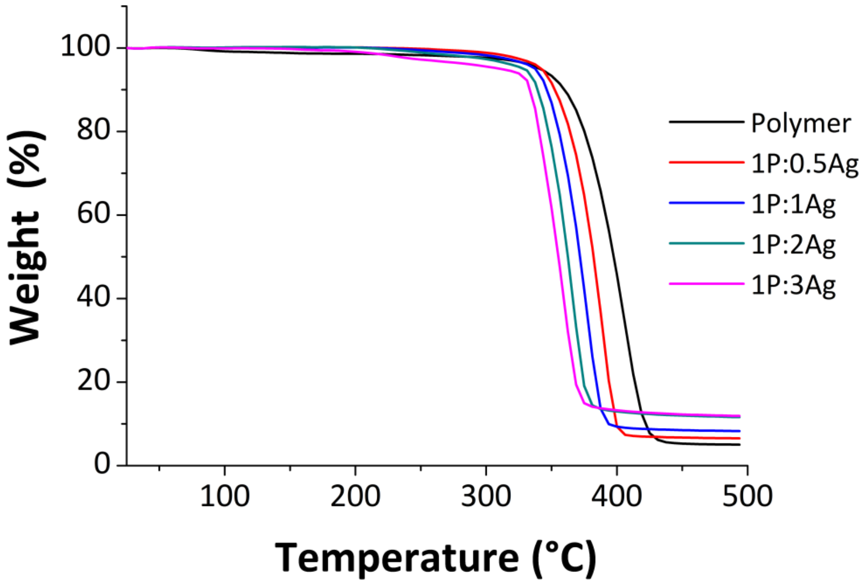

Fig. S18 TGA traces of the polymer **D** and the nanocomposites

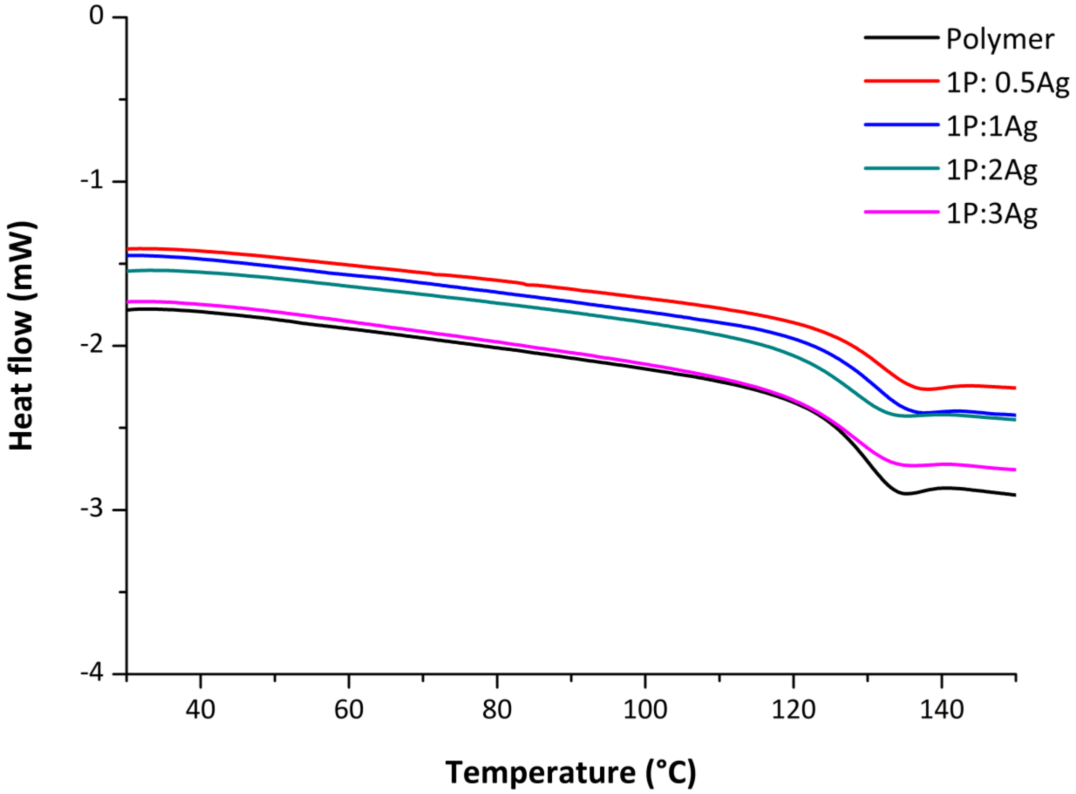

Fig. S19 DSC curves of the polymer **B** and the nanocomposites

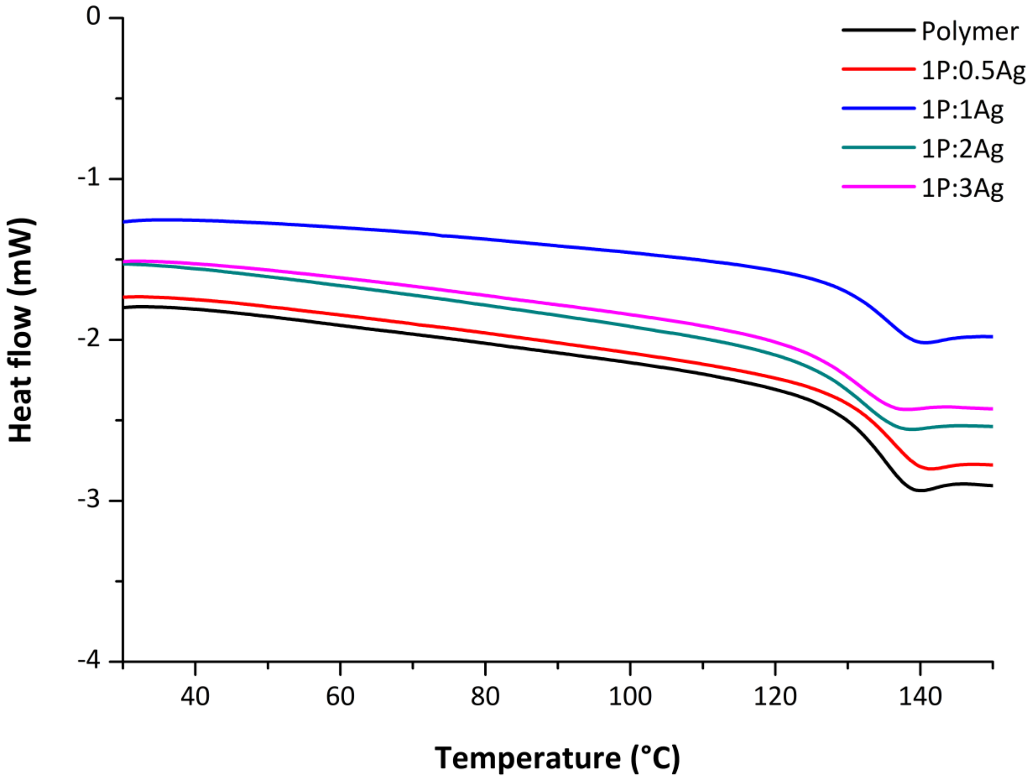

Fig. S20 DSC curves of the polymer **C** and the nanocomposites

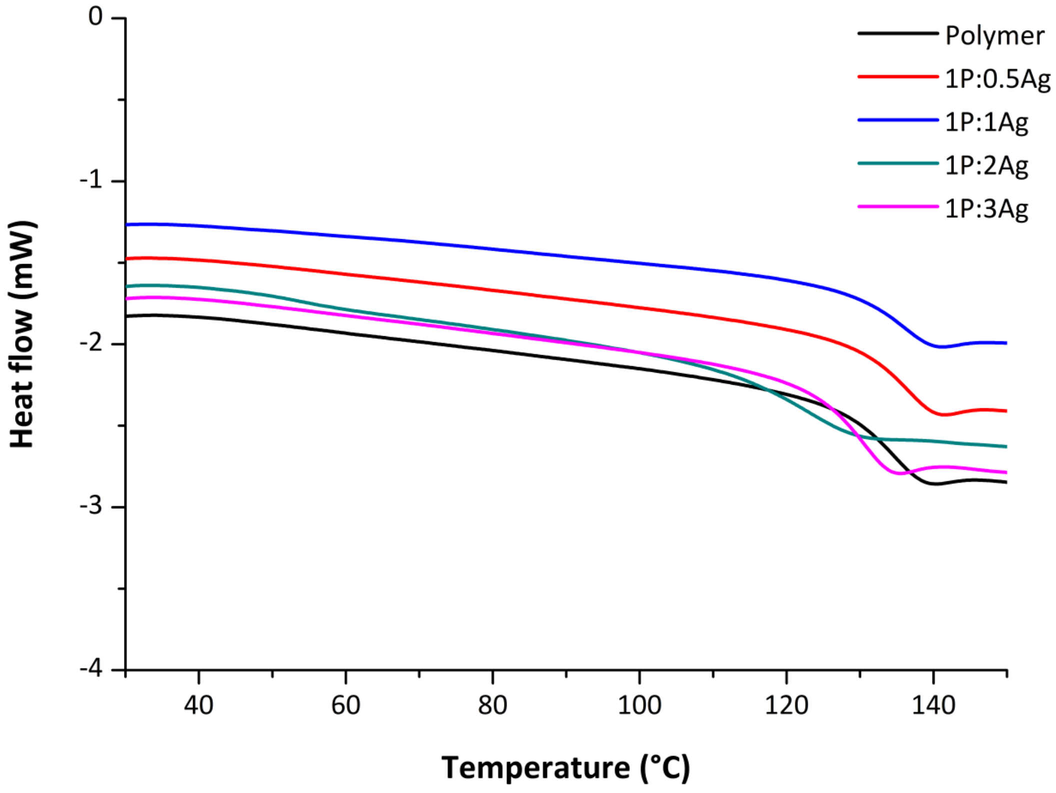

Fig. S21 DSC curves of the polymer **D** and the nanocomposites

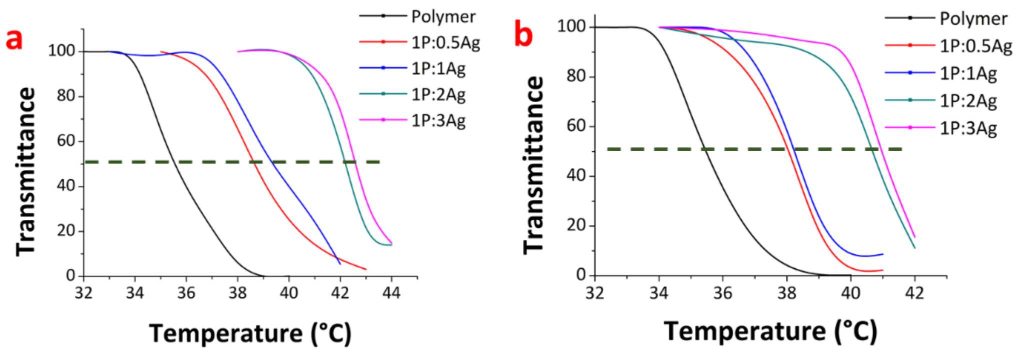

Fig. S22 LCST behaviors of the polymer and the nanocomposites **B** (left) and **C** (right)

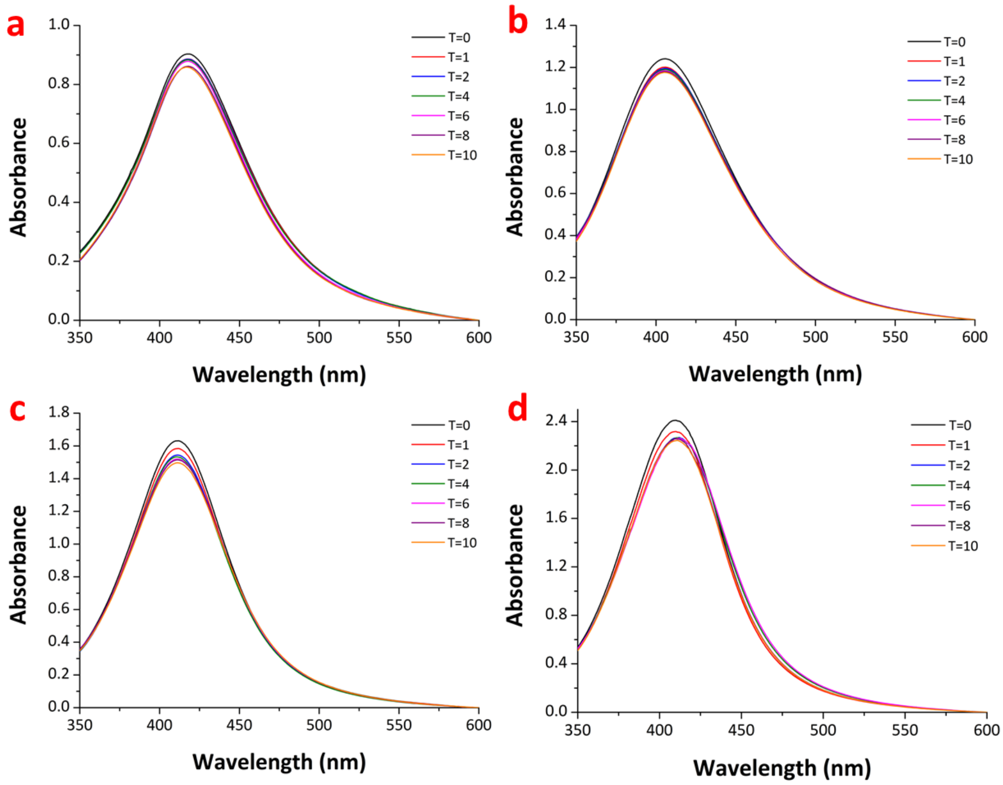

Fig. S23 Stability studies for polymer **A** (up) and **B** (down) with the ratio (**2**) (a, c) and (**3**) (b, d) in H_2O

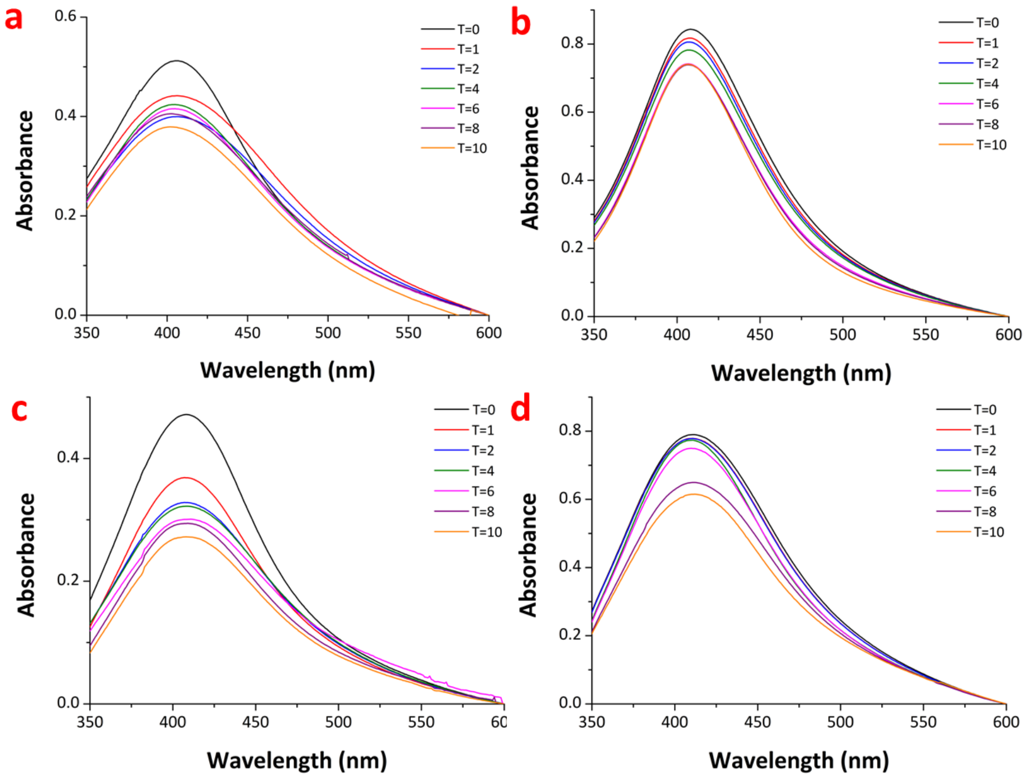

Fig. S24 Stability studies for polymer **C** (up) and **D** (down) with the ratio (**2**) (a, c) and (**3**) (b, d) in H_2O

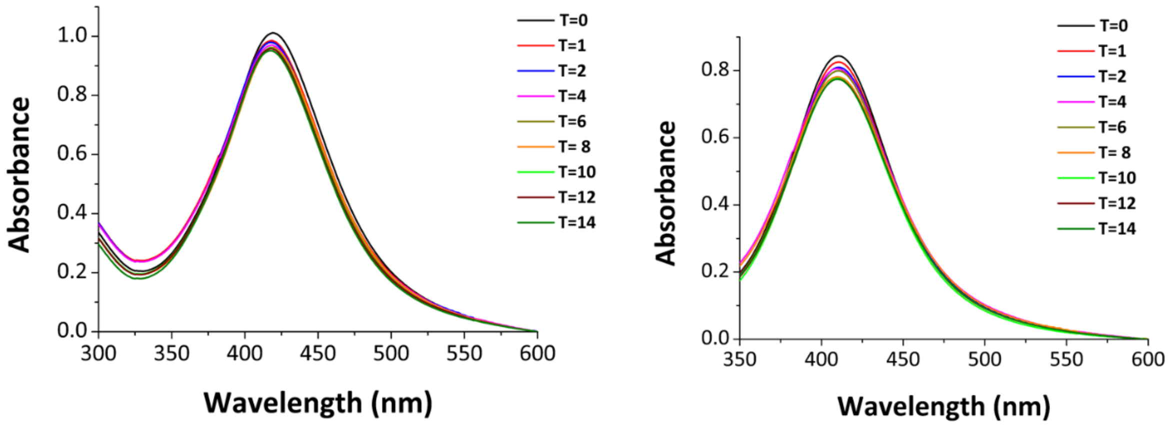

Fig. S25 Stability studies for the ratio (**3**) with polymer **A** (left) and **B** (right) in PBS

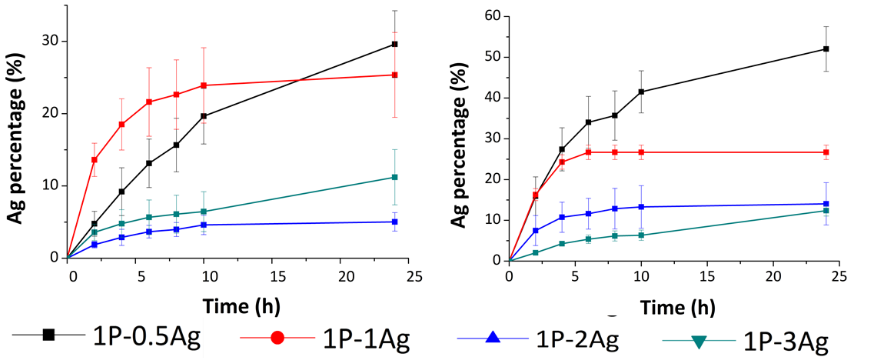

Fig. S26 Ag^+ release kinetics of the nanocomposites **B** at RT (left) and at 37 °C (right)

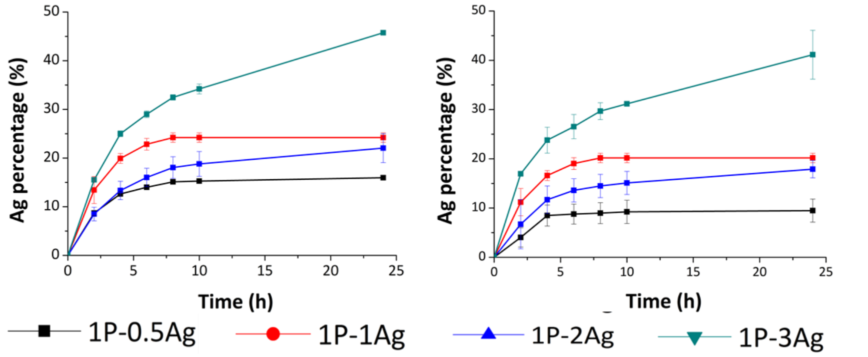

Fig. S27 Ag^+ release kinetics of the nanocomposites **C** at RT (left) and at 37 °C (right)

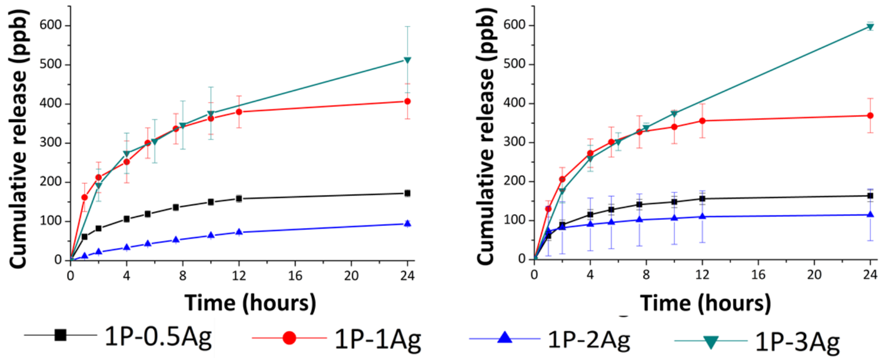

Fig. S28 Ag^+ release kinetics of the nanocomposites **A** at RT (left) and at 37 °C (right)

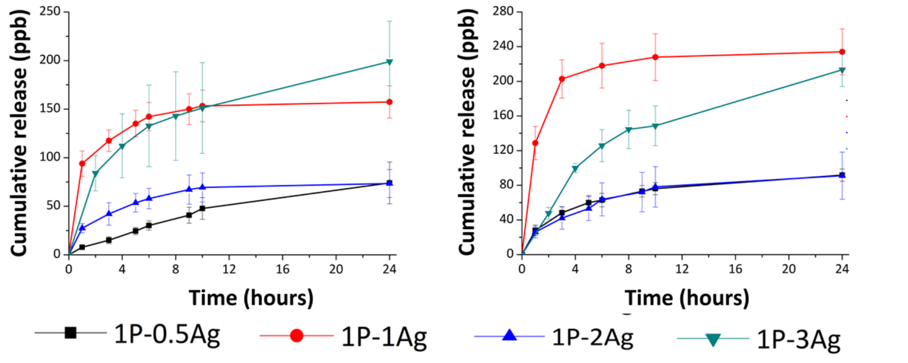

Fig. S29 Release kinetics of the nanocomposites **B** at RT (left) and at 37 °C (right)

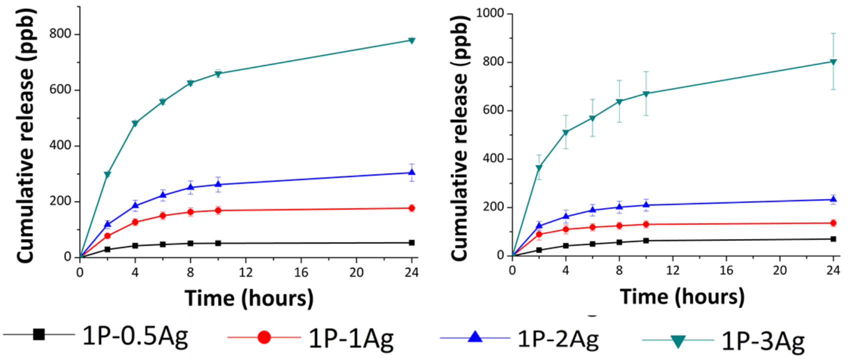

Fig. S30 Ag^+ release kinetics of the nanocomposites **C** at RT (left) and at 37 °C (right)

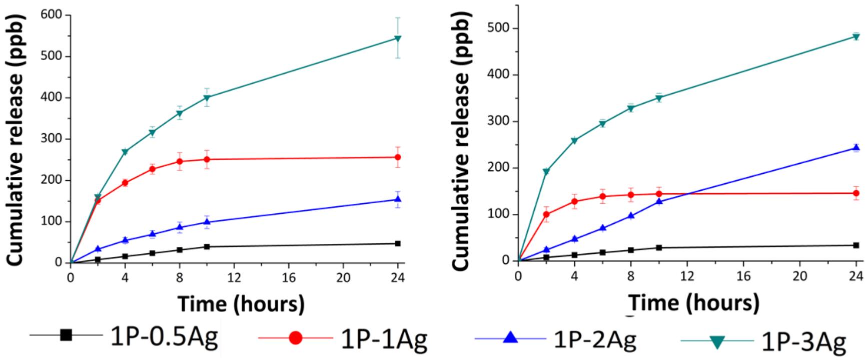

Fig. S31 Ag^+ release kinetics of the nanocomposites **D** at RT (left) and at 37 °C (right)

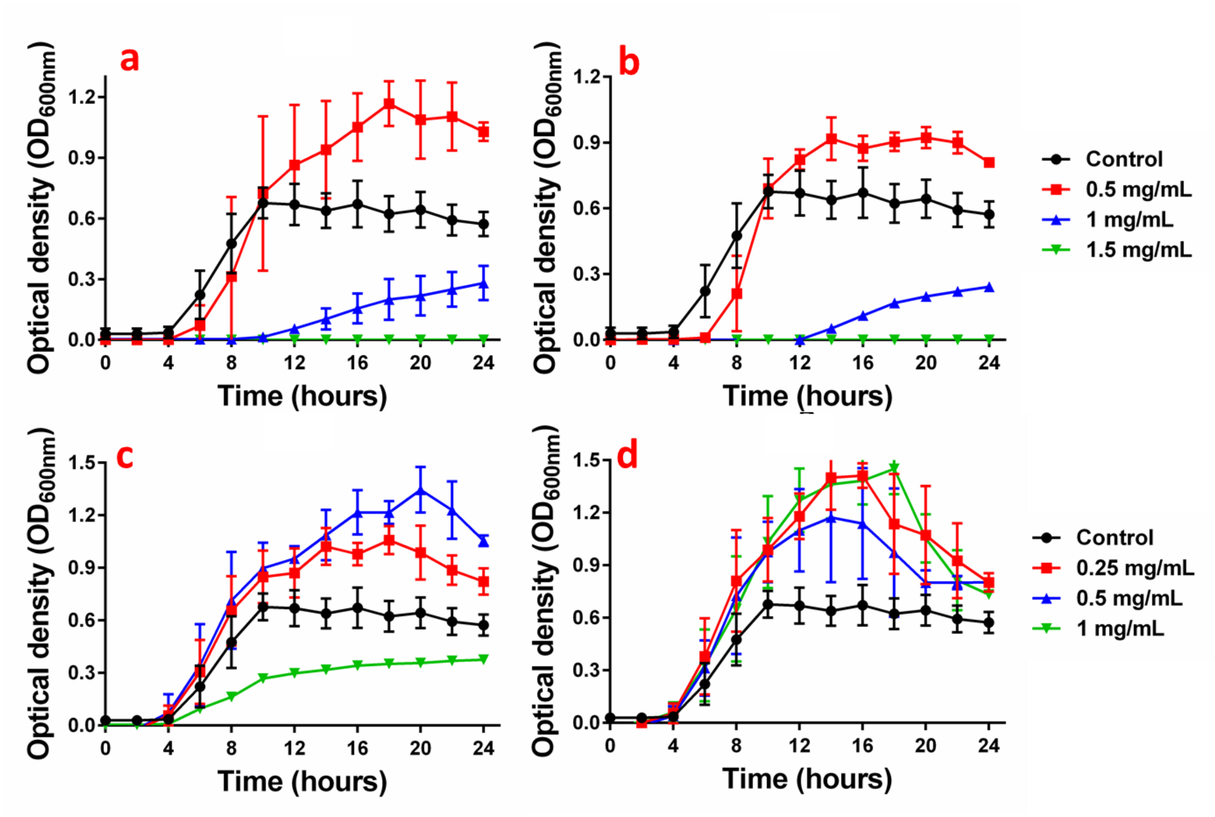

Fig. S32 Growth curves of the nanocomposites **B** with the different ratios (**1**) (a), (**2**) (b), (**3**) (c), (**4**) (d) at various concentrations against *E. Coli*

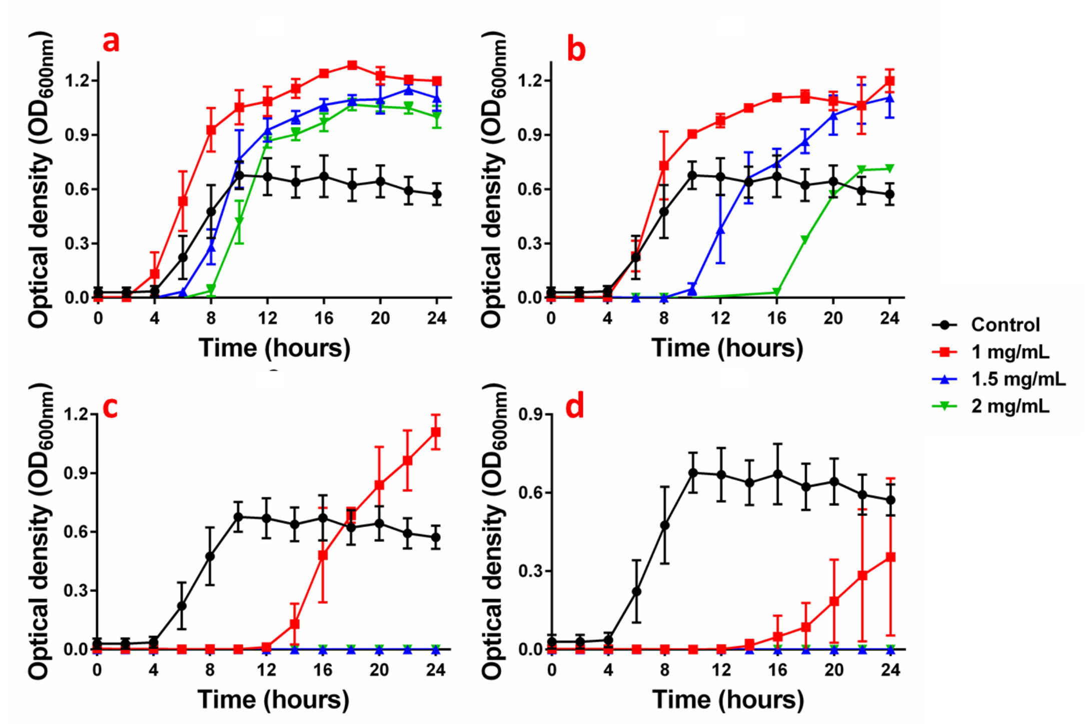

Fig. S33 Growth curves of the nanocomposites **C** with the different ratios (**1**) (a) (**2**) (b), (**3**) (c), (**4**) (d) at various concentrations against *E. Coli*

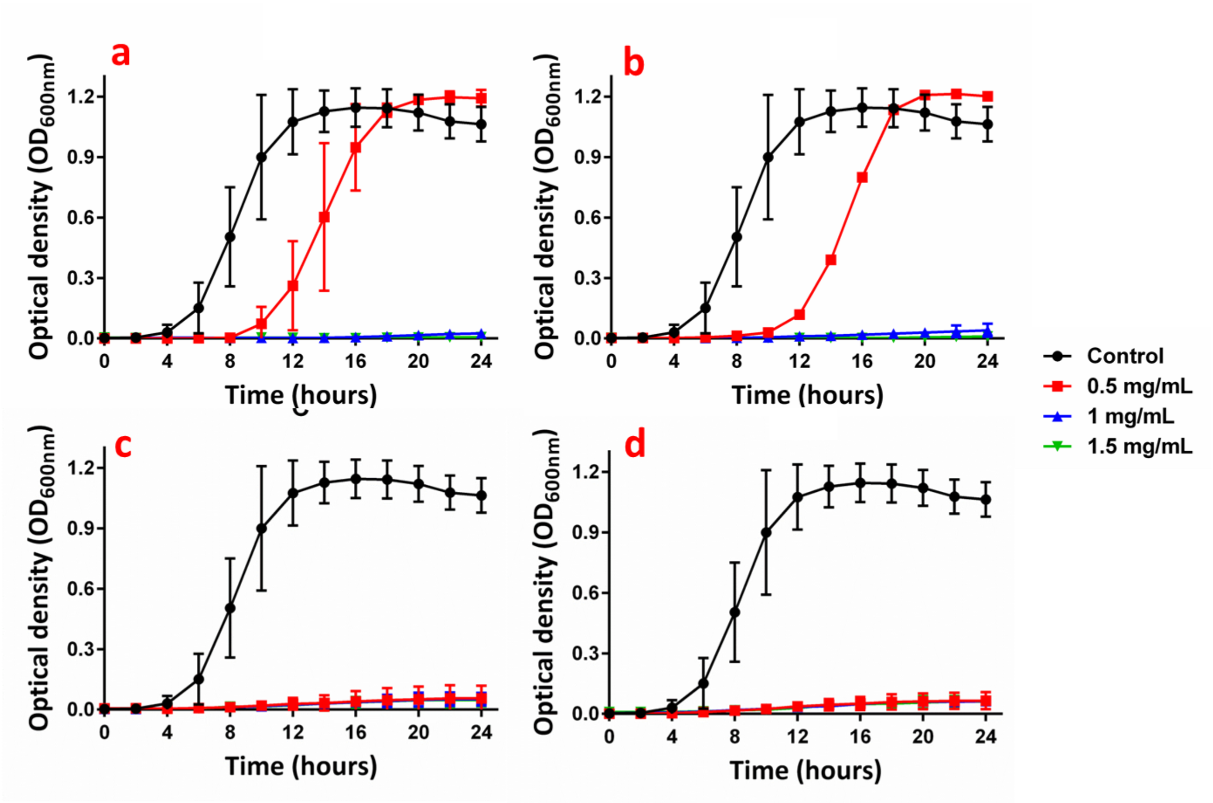

Fig. S34 Growth curves of the nanocomposites **B** with the different ratios (**1**) (a) (**2**) (b), (**3**) (c), (**4**) (d) at various concentrations against *S. aureus*

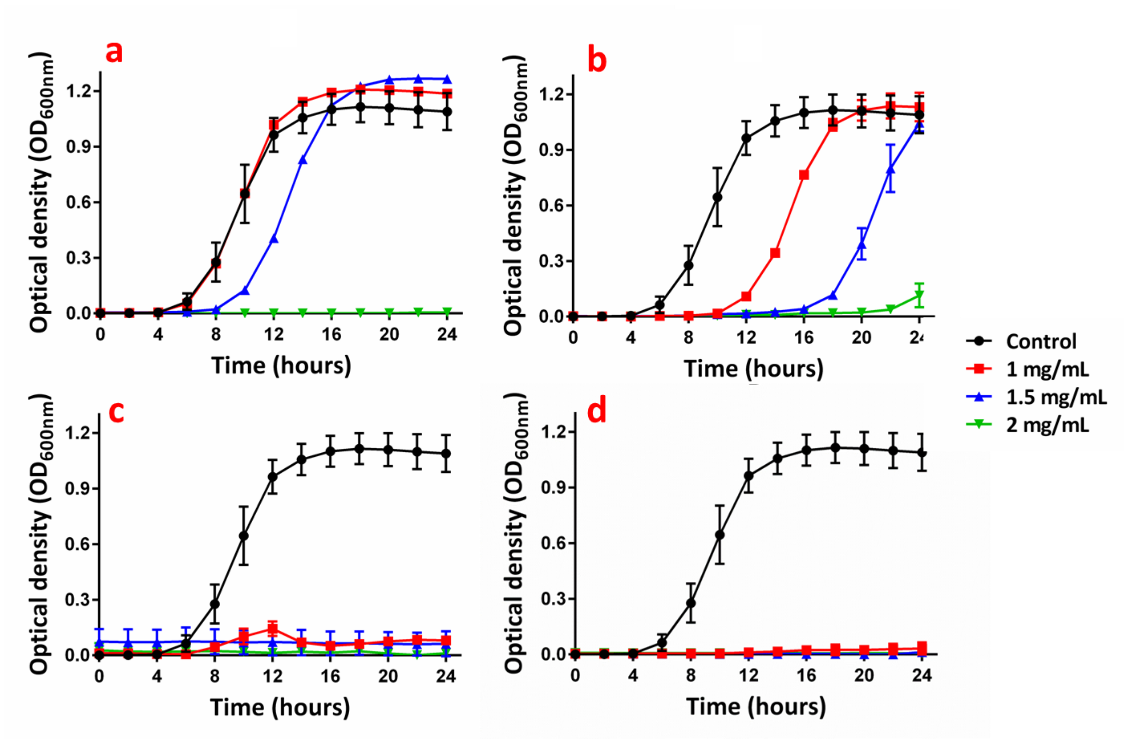

Fig. S35 Growth curves of the nanocomposites **C** with the different ratios (**1**) (a) (**2**) (b), (**3**) (c), (**4**) (d) at various concentrations against *S. aureus*
